# γδ-Enriched CAR-T cell therapy for bone metastatic castrate-resistant prostate cancer

**DOI:** 10.1126/sciadv.adf0108

**Published:** 2023-05-03

**Authors:** Jeremy S. Frieling, Leticia Tordesillas, Xiomar E. Bustos, Maria Cecilia Ramello, Ryan T. Bishop, Junior E. Cianne, Sebastian A. Snedal, Tao Li, Chen Hao Lo, Janis de la Iglesia, Emiliano Roselli, Ismahène Benzaïd, Xuefeng Wang, Youngchul Kim, Conor C. Lynch, Daniel Abate-Daga

**Affiliations:** ^1^ Department of Tumor Biology, H. Lee Moffitt Cancer Center & Research Institute, Tampa, FL, USA.; ^2^ Department of Immunology, H. Lee Moffitt Cancer Center & Research Institute, Tampa, FL, USA.; ^3^ Department of Pathology Research, H. Lee Moffitt Cancer Center & Research Institute, Tampa, FL, USA.; ^4^ Department of Biostatistics and Bioinformatics, H. Lee Moffitt Cancer Center & Research Institute, Tampa, FL, USA.

## Abstract

Immune checkpoint blockade has been largely unsuccessful for the treatment of bone metastatic castrate-resistant prostate cancer (mCRPC). Here, we report a combinatorial strategy to treat mCRPC using γδ-enriched chimeric antigen receptor (CAR) T cells and zoledronate (ZOL). In a preclinical murine model of bone mCRPC, γδ CAR-T cells targeting prostate stem cell antigen (PSCA) induced a rapid and significant regression of established tumors, combined with increased survival and reduced cancer-associated bone disease. Pretreatment with ZOL, a U.S. Food and Drug Administration–approved bisphosphonate prescribed to mitigate pathological fracture in mCRPC patients, resulted in CAR-independent activation of γδ CAR-T cells, increased cytokine secretion, and enhanced antitumor efficacy. These data show that the activity of the endogenous Vγ9Vδ2 T cell receptor is preserved in CAR-T cells, allowing for dual-receptor recognition of tumor cells. Collectively, our findings support the use of γδ CAR-T cell therapy for mCRPC treatment.

## Teaser

Vγ9Vδ2 T cells expressing chimeric antigen receptor (CAR) maintain a functional TCR and induce prostate tumor regression in bone.

## INTRODUCTION

Despite the introduction of novel androgen receptor signaling inhibitors and targeted/chemotherapies, advanced prostate cancer eventually becomes refractory leading to the demise of the patient (*1*, *2*). Of the patients that succumb to the disease, up to 90% will have evidence of bone metastasis (*1*). Bone metastatic castrate-resistant prostate cancer (mCRPC) also causes extensive bone disease, resulting in skeletal-related events such as hypercalcemia, spontaneous fractures, and extreme bone pain, which contribute to cancer morbidity and mortality (*3*). This feature is exacerbated by osteopenia, which is often associated with long-term androgen withdrawal (*4*). To counteract this, nitrogen-containing bisphosphonates (nBPs) such as ZOL are routinely administered to patients with bone mCRPC (*5*). nBPs are taken up by the skeleton and promote the apoptosis of bone-resorbing osteoclasts upon their release by disrupting the mevalonate metabolic pathway, leading to the accumulation of isopentenyl pyrophosphate (IPP) (*6*). Although nBPs prevent pathological fracture and improve patient quality of life, they do not improve overall survival (*7*). Consequently, there is an urgent need for improved therapies with high antitumor potency.

While effective in other cancer settings, immunotherapy success for the treatment of mCRPC has been limited, in part due to low immunogenicity (*8*–*10*). For example, trials with checkpoint blockade inhibitors in metastatic disease have shown minimal effect (*11*–*13*). Recent advances using T cells engineered to express tumor-recognizing chimeric antigen receptors (CAR-T) offer a new means with which to target refractory bone mCRPC (*14*). However, targeting solid malignancies with CAR-T has proved challenging (*15*). Lymphocyte populations expressing predominantly αβ type T cell receptors (TCRs) (αβ T cells) have traditionally been used for CAR-T expression, but here, we hypothesized that the relatively understudied Vγ9Vδ2 T lymphocytes (γδ T cells) represent an attractive vehicle for CAR-T cell therapy given their enhanced cytotoxicity and their ability to recognize phosphoantigens such as IPP, which accumulates in the bone-cancer microenvironment when ZOL is administered (*16*–*19*). Critically for CAR-T efficacy, a reliable and highly expressed tumor antigen needs to be identified. In this regard, more than 90% of prostate cancers express prostate stem cell antigen (PSCA), with even higher positivity (>99%) noted in bone metastatic disease (*20*, *21*). PSCA expression is also significantly lower in normal prostate tissue, minimizing the potential for “on target, off tumor” effects (*20*). In the present study, we demonstrate the efficient expression of an anti-PSCA CAR expressed in human γδ T cells expanded from peripheral blood and their potent cytotoxicity against bone mCRPC cells in vivo. Furthermore, we show that this effect is augmented by the nBP, ZOL. We observed no overt toxicities in tumor-bearing mice and that the anti-PSCA γδ CAR-T treatment significantly reduced cancer-associated bone disease. Together, the data reveal that γδ T cell–based CAR therapies effectively mitigate bone metastatic prostate tumors and that the infusion of anti-PSCA γδ CAR-T cells in bone metastatic prostate cancer patients has the potential to be highly effective, due to the preexisting therapeutic application of ZOL in this patient population.

## RESULTS

### Bone metastatic prostate tumors express ligands of endogenous γδ TCRs

**Fig. 1.**
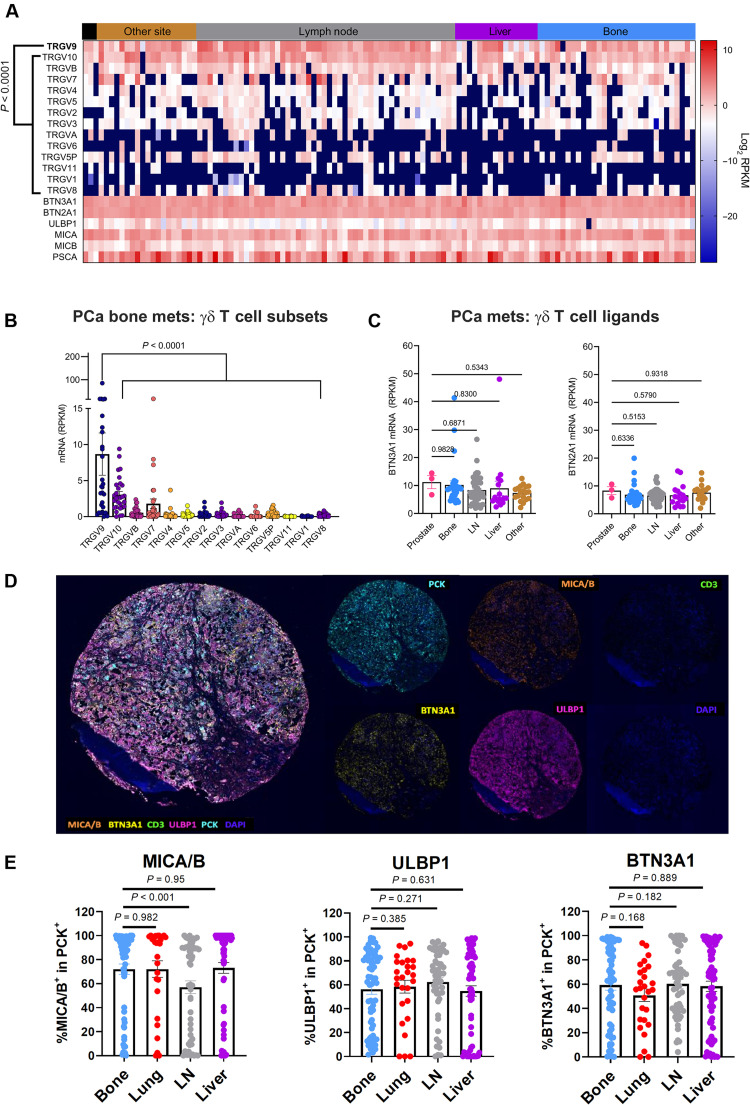
BTN expression and spontaneous gamma/delta T cell infiltration in prostate tumors. (**A**) Bioinformatic analysis of TCR gamma variable regions (*TRGV*), *BTN3A1*, *BTN2A1*, *ULBP1*, *MICA*, *MICB*, and *PSCA* in bone and soft tissue biopsies from 118 individuals with mCRPC (SU2C/PCF dataset). (**B**) Specific comparison of *TRGV* expression in bone metastases. RPKM, reads per kilobase of transcript per million mapped reads. (**C**) *BTN2A1* and *BTN3A1* expression in bone, liver, lymph node (LN), and other soft tissue metastasis sites compared to primary prostate tumors. (**D**) Representative multiplex staining of bone metastasis sample from TMA containing 1-mm cores. (**E**) Expression of BTN3A1 and stress markers MICA/B and ULBP1 in tumor cells (PCK^+^) from a TMA containing samples from patients with metastatic prostate cancer in different tissues. Only samples with more than 100 PCK^+^ cells were considered for the analysis (*n* = 64, 27, 52, and 70 for bone, lung, lymph node, and liver samples, respectively). Each dot represents a sample. Bars represent means ± SEM. Linear mixed effect model was used for statistical analysis.

γδ T cells play a role in both the innate and the adaptive immune system and represent 1 to 10% of T cells in peripheral blood (*22*). They have been associated with tumor immunity, and different subsets of γδ T cells have been described (*23*). To determine whether γδ T cells could potentially infiltrate prostate tumors, we analyzed the SU2C/PCF dataset that contained metastatic bone and soft tissue tumor biopsies from 118 mCRPC individuals (*24*) to measure expression levels of 14 TCR-γ genes (*TRGV9*, *TRGV10*, *TRGVB*, *TRGV7*, *TRGV4*, *TRGV5*, *TRGV2*, *TRGV3*, *TRGVA*, *TRGV6*, *TRGV5P*, *TRGV11*, *TRGV1*, and *TRGV8*). Our analyses identified that *TRGV9* transcripts were highly expressed compared to other TRGV genes, suggesting that Vγ9Vδ2 T cells may be enriched in prostate cancer, and particularly in bone metastases (Fig. 1, A and B). Analysis of an independent dataset [The Cancer Genome Atlas Prostate Adenocarcinoma (TCGA-PRAD)] containing 498 primary tumor samples also confirmed these findings regarding *TRGV9* (fig. S1A).

γδ T cells express several endogenous receptors that mediate the recognition of infected and stressed cells. More specifically, the Vγ9Vδ2 TCR can react to the accumulation of phosphoantigens that may occur spontaneously in tumor cells presenting a dysregulated mevalonate pathway (*23*), or because of the pharmacological inhibition of the mevalonate pathway with reagents such as ZOL. TCR-mediated reactivity against phosphoantigen accumulation depends on the expression of butyrophilins (BTN3A1 and BTN2A1) by the target cells. In our analysis of the SU2C/PCF Dream Team dataset, we found *BTN3A1* and *BTN2A1* transcripts to be expressed in bone metastatic tumors at similar levels to prostate tumors and soft tissue metastases to the liver and lymph nodes (Fig. 1C). In addition to signaling through butyrophilins, γδ T cells express activating natural killer receptors, including NKG2D, that bind to stress-inducible surface molecules in target cells, such as ULBP1, MICA, and MICB (*22*). This recognition can induce or further enhance immune attack by γδ T cells. Notably, we observed the expression of *ULBP1*, *MICA*, and *MICB* transcripts in bone metastatic tumors (fig. S1B). We also detected a trend toward the heightened expression of *PSCA* in metastatic tumor sites, including bone, relative to primary prostate tumors (fig. S1B).

Next, we assessed the expression of γδ T cell endogenous receptor ligands in metastatic prostate cancer specimens. To this end, multiplex staining was performed on tumor microarrays (TMAs) containing cores from 45 patients with metastasis in bone, liver, lymph nodes, and lung (Fig. 1, D and E; fig. S1, C to E; and table S1). Of the full cohort of samples, 213 cores presented more than 100 pan-cytokeratin positive (PCK^+^) cells, enabling further analysis. Gating on PCK^+^ cells as a marker for tumor cells (fig. S1D), bone metastatic samples presented high expression of BTN3A1 in most of the cores, with the majority of cancer cells (≥50%) staining positively for BTN3A1 expression in 63% of the cores analyzed (Fig. 1E). Likewise, MICA/B and ULBP1 were abundantly expressed, with more than 75 and 57% of the bone metastatic samples showing the expression of these markers in more than 50% of the tumor cells, respectively. Moreover, prior bisphosphonate therapy did not decrease the expression of any of the ligands analyzed (fig. S1E). We also noted that high expression of BTN3A1 and NKG2D ligands was maintained in liver, lymph node, and lung metastases (Fig. 1E). But in contrast to bone, we observed an increased expression of MICA/B, ULBP1, and BTN3A1 in lung and liver samples from patients previously treated with bisphosphonates (fig. S1E). These findings led us to posit that the high expression of these ligands may allow γδ CAR-T cells, as opposed to αβ CAR-T cells, to recognize tumor cells through endogenous receptors, thus enhancing their cytolytic potential. We further hypothesized that γδ CAR-T cells may benefit from their use in combination with zoledronate (ZOL), or other agents that induce accumulation of phosphoantigens.

### γδ T cells expressing CD28-costimulated second-generation CARs display maximum IFN-γ secretion and targeted prostate cancer cell cytotoxicity

**Fig. 2.**
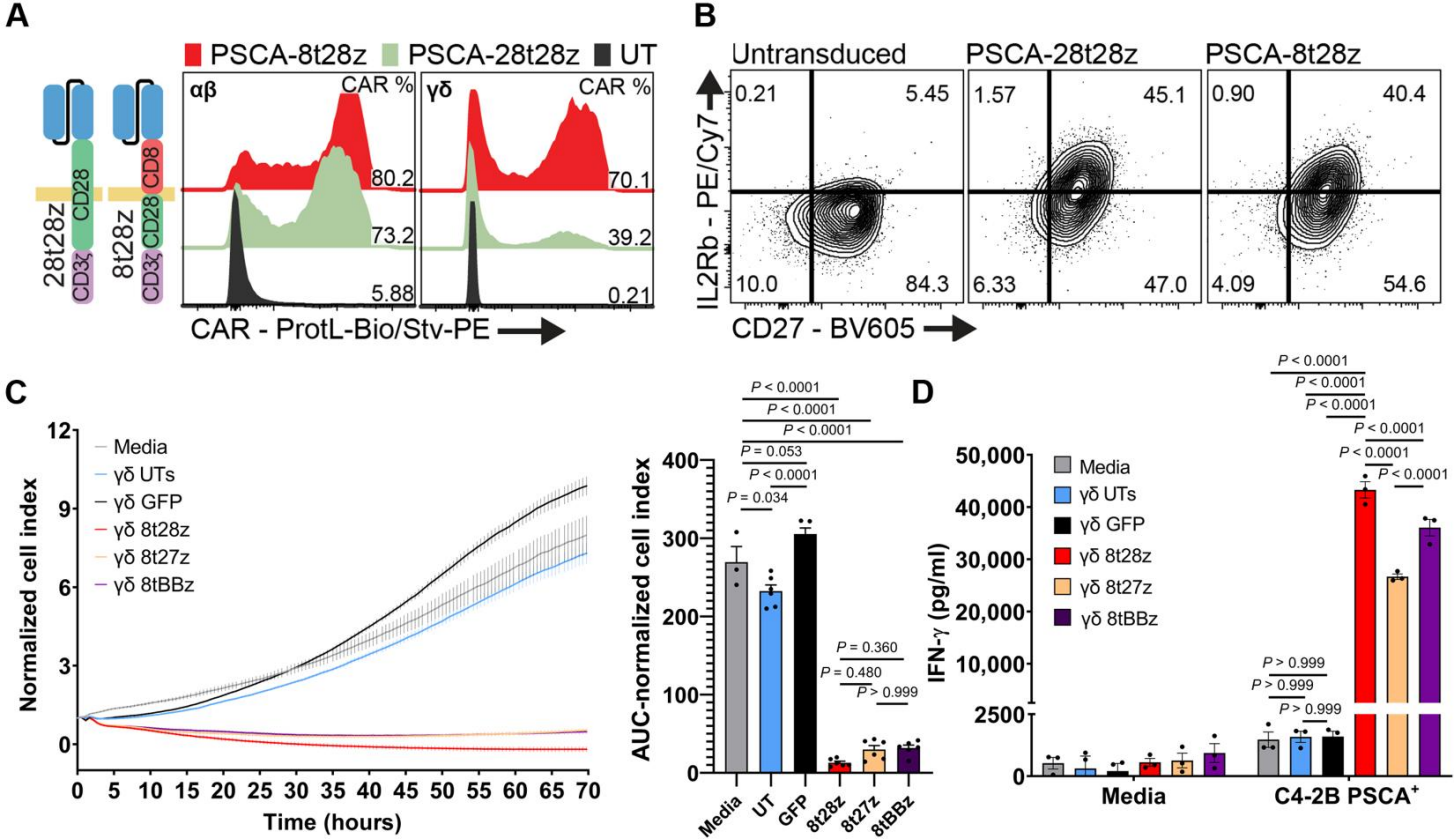
Impact of CAR endodomain design. (**A**) Representative plot of the comparison of CAR expression in αβ versus γδ T cells from the same donor. Surface CAR expression assessed by flow cytometry after staining with Biotin-Protein L and streptavidin-PE. Gated on lymphoid, single, viable cells. (**B**) Representative plot of the phenotypic changes induced in γδ T cells by expression of CARs with different CAR hinge/transmembrane domains (CD8 or CD28). Gated on lymphoid, single, viable (DAPI^−^), CD3^+^CD45^+^ , Vδ2 TCR T cells. (**C**) Real-time cytotoxicity assay (RTCA) analysis of C4-2B PSCA^+^ cells cocultured in the absence of T cells (Media), with UT γδ T cells, GFP-expressing γδ T cells, or γδ CAR-T cells (ratio 1:1 tumor:T cells), displaying different costimulatory domains (CD27, CD28, and 4-1BB) and the same transmembrane domain (CD8). Bars represent means ± SEM. Quantification of AUC is shown as bar graph with statistically significant differences calculated by one-way ANOVA with multiple comparisons. (**D**) Quantification of IFN-γ (ELISA) in supernatants from different γδ CAR-T cells cocultured with C4-2B PSCA^+^ cells overnight. Percentage of CAR^+^ cells was normalized across samples. Data from a representative donor of *n* = 3 are shown. Each dot represents a technical replicate. Statistically significant differences were calculated by two-way ANOVA with multiple comparisons.

To generate γδ CAR-T cells, we first transduced Vγ9Vδ2-enriched T-cells with a second-generation, CD28-costimulated CAR construct (28t28Z) that has previously shown antitumor efficacy against PSCA-expressing tumors, when transduced into αβ T cells (*25*). In parallel, αβ T cells were transduced to serve as a control. While this CAR was expressed in a sizable proportion of the αβ T cells, its expression was much lower in γδ T cells derived from the same donor (Fig. 2A, gating strategy of γδ shown in fig. S2). However, we observed a substantial improvement in γδ T cell expression of a modified CAR where the CD28-derived hinge and transmembrane domains (but neither of the signaling moieties) are replaced with a CD8α-derived hinge/transmembrane (8t28Z) (Fig. 2A). Although we observed a slight difference in titers of the viral vectors (fig. S3A), it is unlikely that this is the sole explanation for the lower expression of the −28T28Z CAR observed across donors (fig. S3, B and C) because the number of integrated copies of the vector was comparable among the different constructs (fig. S3D). γδ T cells expressing either of these CARs showed greater expression of the β subunit of the interleukin-2 receptor (IL-2Rβ), as previously described for αβ CAR-T cells (Fig. 2B) (*26*). These results indicate that CAR designs that are suitable for αβ T cells are not necessarily optimal for use in their γδ counterparts, and that slight modifications of the structural elements can greatly enhance CAR expression.

Using C4-2B prostate cancer cells expressing PSCA, we next tested the cytotoxic activity of the 8t28Z CAR in comparison to two variations where the costimulatory domain was replaced with either a CD27- or 4-1BB–derived moiety (8t27Z and 8tBBZ, respectively). Both untransduced (UT) and mock-transduced [green fluorescent protein (GFP)] T cells were used as negative controls. Real-time analysis of tumor cell adherence (which we used as a surrogate of tumor cell viability) demonstrated that the addition of γδ CAR-T cells resulted in a significant reduction of C4-2B PSCA^+^ cell viability, whereas no effect was observed with the control groups (Fig. 2C). Notably, no significant differences in the area under the curve (AUC; used as a surrogate of cumulative viability) were observed among the γδ CAR-T–treated groups (Fig. 2C and fig. S3, E and F). However, quantitation of interferon-γ (IFN-γ) secretion following overnight culture showed that the 8t28Z design was superior to other variations analyzed (Fig. 2D and fig. S3G), and this difference was not attributable to transduction efficiency (fig. S3D). We therefore selected the 8t28Z design for further analysis, based on prior reports indicating that IFN-γ secretion can modulate the tumor microenvironment of solid tumors and increase the efficacy of CAR-T cells (*27*, *28*).

### γδ-enriched CAR-T cells induce regression of intratibial prostate tumors

**Fig. 3.**
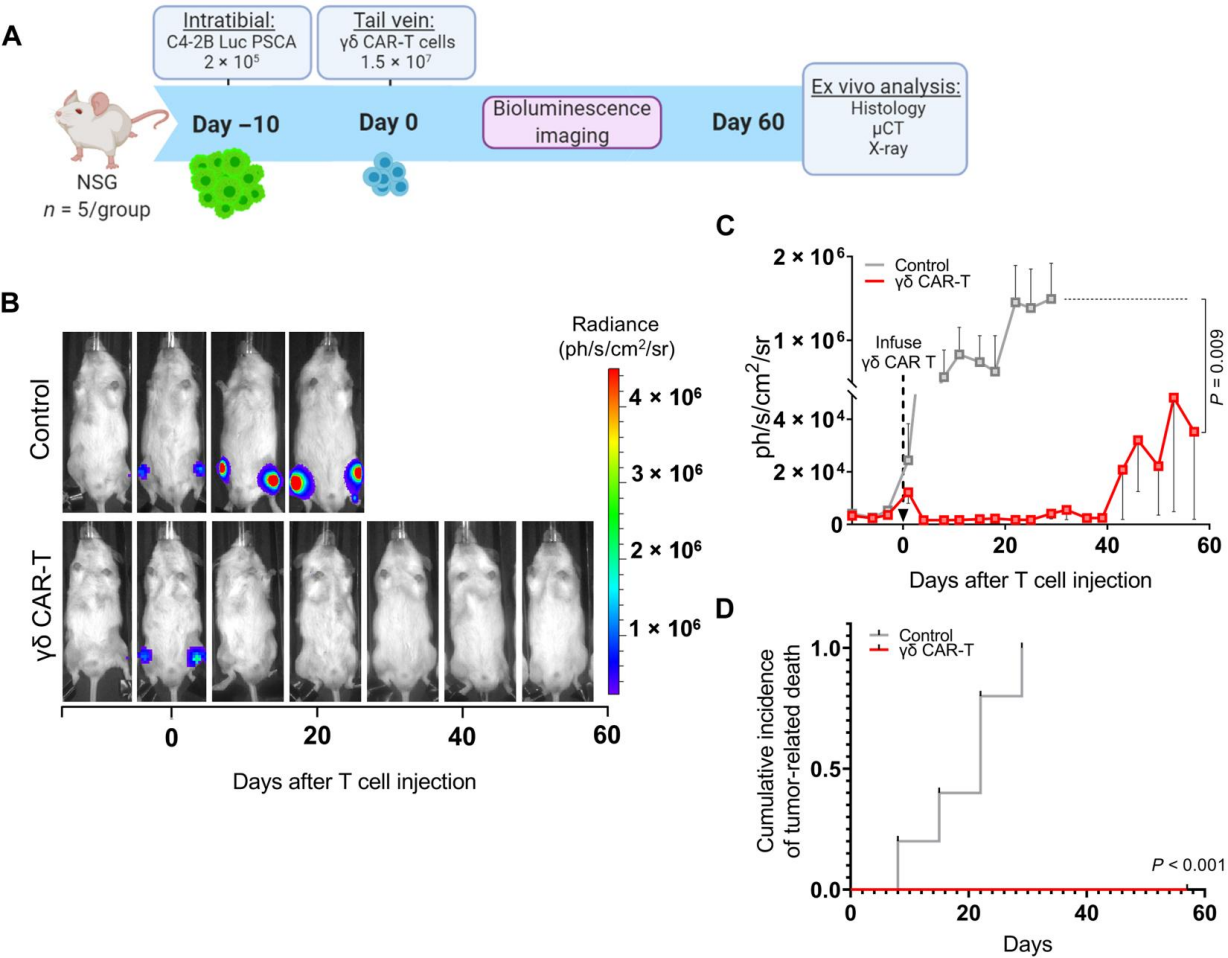
Regression of intratibial prostate tumors induced by γδ-enriched CAR-T cells. (**A**) Schematic of in vivo experimental design to test γδ CAR-T cells using an intratibial injection model of bone metastatic prostate cancer. (**B**) Representative bioluminescence images from control (untreated) and γδ CAR-T–treated C4-2B PSCA^+^ tumor-bearing mice. (**C**) Quantification of tumor bioluminescence presented as mean luminescence (photons s^−1^ cm^−2^ sr^−1^) from control and γδ CAR-T–treated C4-2B PSCA^+^ tumor-bearing mice (*n* = 5 mice per group). γδ CAR-T cells were delivered on day 0 as indicated by an arrow. The *y* axis is split to allow visibility of recurring tumors in γδ CAR-T–treated mice at late stages; see fig. S4 for same data graphed with the solid *y* axis. (**D**) Competing risk survival analysis comparing γδ CAR-T–treated C4-2B PSCA^+^ tumor-bearing mice to untreated control.

To assess the antitumor activity of anti-PSCA γδ 8t28Z CAR-T cells in vivo, PSCA-expressing C4-2B prostate cancer cells (2.0 × 10^5^ per injection) were inoculated into both the left and right tibias of 6-week-old male NSG (NOD.Cg-Prkdcscid Il2rgtm1Wjl/SzJ) mice (*n* = 10) and monitored via bioluminescence imaging as a readout for tumor burden (Fig. 3A). After 10 days, mice were randomized into two groups based on luminescence signal, with one cohort serving as an untreated control and the other receiving anti-PSCA γδ CAR-T cells (1.5 × 10^7^) via tail vein inoculation. Subsequent bioluminescent imaging revealed that mice treated with anti-PSCA γδ CAR-T therapy demonstrated a rapid and significant regression of tumors (*P* = 0.009) within 24 hours that was sustained up to 30 days after T cell transfer (Fig. 3, B and C). At later time points, we noted recurrences in two of the five anti-PSCA γδ CAR-T–treated mice but we observed the growth of these tumors to be much slower compared to untreated control tumors (fig. S4). The study endpoint was 1 × 10^6^ relative light units (RLUs), and consistent with growth data, we observed significantly decreased hazard risk of death in comparison to γδ CAR-T–treated mice to untreated control (*P* < 0.001) (Fig. 3D). No overt toxicities (weight loss/lethargy/hunched posture) were noted in the γδ CAR-T–treated group. Collectively, these findings indicate that anti-PSCA γδ CAR-T cells can significantly drive the regression of established bone mCRPC in vivo.

### Treatment with γδ-enriched CAR-T cells mitigates tumor-induced bone disease

**Fig. 4.**
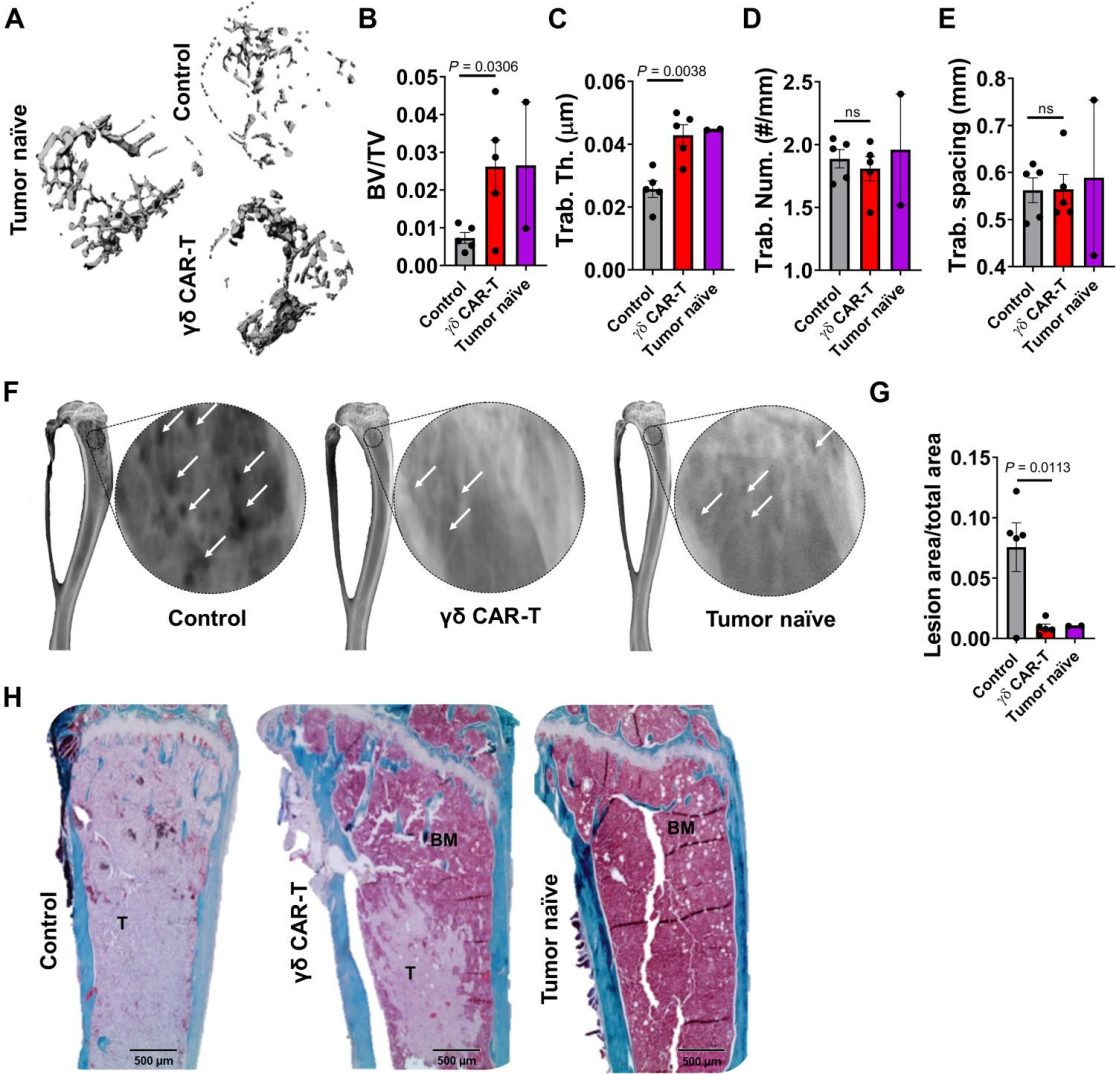
Mitigation of tumor-induced bone disease by γδ-enriched CAR-T cells. (**A**) Three-dimensional reconstructions of trabecular bone (top-down view) from high-resolution μCT scanning of tumor-naïve, control (untreated), and γδ CAR-T cell–treated mice bearing C4-2B PSCA^+^ tumors. (**B** to **E**) Trabecular bone volume, trabecular thickness, trabecular number, and trabecular spacing measurements derived from μCT bone reconstructions. (**F**) Representative x-ray images illustrating osteolytic lesions (white arrows) in tibias from tumor-naïve, untreated control, and γδ CAR-T cell–treated mice. (**G**) Quantification of osteolytic lesion area from x-ray images, presented as ratio to total bone area. (**H**) Representative images of trichrome-stained paraffin-embedded tibia sections. Aquamarine staining indicates bone, light pink indicates tumor (labeled “T”), and dark pink indicates bone marrow (labeled “BM”). *n* = 2 for naïve and 5 for control and γδ CAR-T groups, respectively. Each dot represents a sample. Statistical analyses were performed by two-tailed *t* test, unpaired, between independent groups.

Next, we determined the impact of γδ CAR-T cell therapy on bone metastatic prostate cancer–induced bone disease. We performed ex vivo high-resolution micro–computed tomography (μCT) on isolated tibias from study mice and observed significantly higher trabecular bone volume and trabecular thickness in the bones of mice treated with anti-PSCA γδ CAR-T, via regression of prostate cancer cells (Fig. 4, A to E). Ex vivo x-ray analysis also identified significantly less osteolysis in the anti-PSCA γδ CAR-T–treated group compared to the controls (Fig. 4, F and G). Trichrome staining of intratibial tumor sections confirmed our in vivo imaging observations and showed either no evidence of (*n* = 3 of 5) or greatly reduced (*n* = 2 of 5) tumor burden in the bone marrow compartments of anti-PSCA γδ CAR-T–treated mice compared to controls (Fig. 4H). Together, these data indicate that anti-PSCA γδ CAR-T significantly protects against tumor-induced bone disease.

### γδ CAR-T cells maintain a functional endogenous TCR

**Fig. 5.**
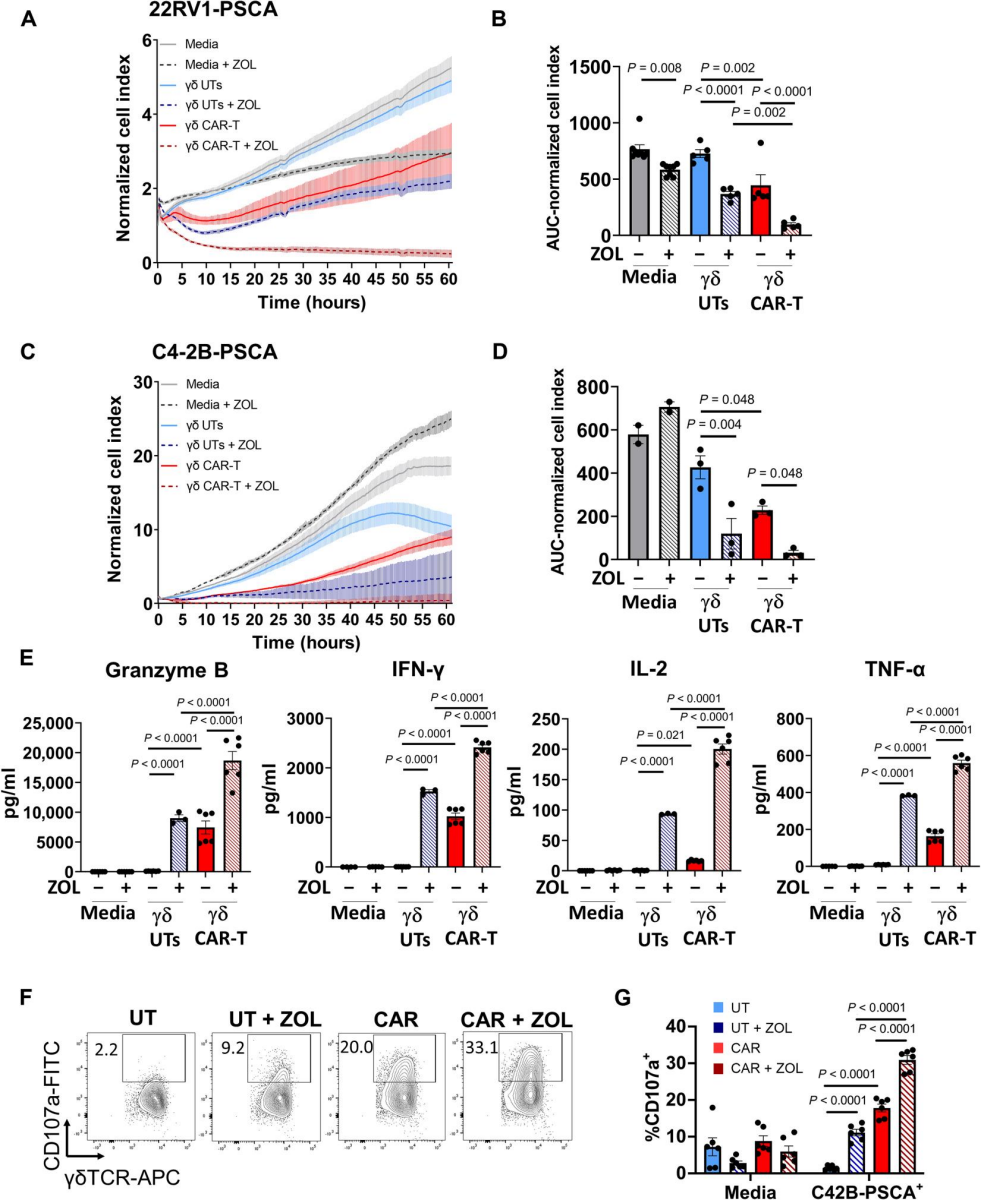
Dual recognition of tumor by γδ CAR-T cells. (**A**) RTCA analysis of 22Rv1 PSCA^+^ cells, cocultured with γδ UT cells or anti-PSCA γδ CAR-T cells (ratio 1:2.5 tumor:T cells), in the presence or absence of 4 μM ZOL. (**B**) Quantification of AUC of the RTCA analysis shown in (A). (**C**) RTCA analysis of C4-2B PSCA^+^ cells (ratio 1:2.5 tumor:T cells), cocultured with UT γδ T cells or anti-PSCA γδ CAR-T cells, in the presence or absence of 4 μM ZOL. (**D**) Quantification of AUC of the RTCA analysis shown in (C). (**E**) Cytokine release upon coculture of C4-2B PSCA^+^cells with medium or the indicated T cell effectors (ratio 1:5 tumor:T cells) in the presence of 4 μM ZOL. Data from one representative donor of *n* = 3 are shown as means ± SEM. Each dot represents a technical replicate. Statistical analyses were performed by one-way ANOVA, Holm-Šídák test. (**F**) Representative dot plots of CD107a expression in γδ T cells after 24 hours of coculture with C4-2B PSCA^+^ cells. Cells were gated on lymphoid, single, viable, CD45^+^, CD3^+^, TCRγ/δ ^+^. (**G**) Expression (%) of CD107a in γδ T cells cocultured with C4-2B PSCA^+^ for 24 hours. Graphs show data from three different donors. Statistical analyses were performed by two-way ANOVA.

Because the Vγ9Vδ2 TCR is activated by accumulation of phosphoantigens induced by bisphosphonates such as ZOL (*19*), we tested whether γδ CAR-T cells maintained the functionality of their endogenous TCR. To this end, we monitored tumor cell viability over time following exposure to γδ CAR-T cells or control UT γδ T cells in the presence or absence of 4 μM ZOL, the concentration used for γδ T cell expansion ex vivo (*18*, *29*). C4-2B PSCA^+^ and 22Rv1 PSCA^+^ cells were used as target cells, as they both express CD277 (BTN3A1/2/3) (fig. S5). Results show that 22Rv1 PSCA^+^ cells experienced a significant reduction in growth following the addition of either γδ CAR-T cells alone or UT γδ T cells plus ZOL. When γδ CAR-T cells were added in combination with ZOL, a rapid reduction in tumor cell viability was observed and produced the maximum cytotoxic effect (Fig. 5A and fig. S6, A and B). Analysis of cumulative viability represented as AUC of normalized cell index confirmed that the combination of γδ CAR-T cells with ZOL induced the maximum reduction in tumor viability and was significantly different from that achieved by γδ CAR-T cells alone (*P* < 0.001) or UT γδ T cells plus ZOL (*P* = 0.002; Fig. 5B). Although ZOL alone appeared to have a direct effect on tumor cells (*P* < 0.05), a phenomenon that has been previously reported (*30*–*32*), this effect accounted for only a small fraction of the reduction in cell viability. Comparable results were observed when C4-2B PSCA^+^ cells were used as target; however, in this case, ZOL alone did not reduce tumor cell viability (Fig. 5, C and D, and fig. S7, A and B). For reference, we included anti-PSCA αβ CAR-T cells, which showed similar cytotoxic activity as the γδ CAR-T condition but noted that αβ T cells did not benefit from the addition of ZOL (fig. S7, A and B). These data confirmed that ZOL-induced activation is an exclusive feature of γδ T cells. Last, we tested the ability of the endogenous TCR to induce cytokine secretion independently or in concert with CAR activation. C4-2B PSCA^+^ cells were cocultured overnight with γδ CAR-T cells or control γδ UT-T cells, either in the presence or absence of 4 μM ZOL. The concentration of granzyme B, IFN-γ, IL-2, and tumor necrosis factor–α (TNF-α) in the supernatants was quantified using Biotechne's ELLA^TM^ assay. For all cytokines, the addition of ZOL significantly induced cytokine secretion by γδ UT-T cells but this effect was significantly higher in γδ CAR-T cells (Fig. 5E and fig. S7C). Similar results were observed when cells were cocultured overnight with 22Rv1 PSCA^+^ (fig. S6C). Moreover, when γδ CAR-T cells were cultured with unmodified C4-2B or 22Rv1, the addition of ZOL resulted in increased killing of tumor cells (fig. S8), suggesting that γδ CAR-T cells benefit from the presence of ZOL even in the context of antigen heterogeneity. These results indicate that the endogenous γδ TCR remains functional in γδ CAR-T cells and can boost activation induced by the CAR in the presence of ZOL. To test if addition of ZOL increased the activation of γδ T cells, CD107a expression was assessed in γδ T cells after coculture with C4-2B PSCA^+^ for 24 hours. As expected, γδ CAR-T cells demonstrated increased surface expression of CD107a when cultured with PSCA-expressing cells, in comparison with γδ UT cells (Fig. 5, F and G). We also observed that the expression of CD107a was further increased by addition of ZOL, in both γδ CAR-T and γδ UT, indicating that ZOL enhances degranulation of γδ CAR-T cells (Fig. 5G). Collectively, these findings suggest that the TCR in γδ CAR-T cells is functional and that the ability to recognize target cells via two independent mechanisms may increase their antitumor efficacy by maximizing their cytotoxic function.

### ZOL enhances the rate of regression of intratibial prostate tumors induced by γδ-enriched CAR-T cells

**Fig. 6.**
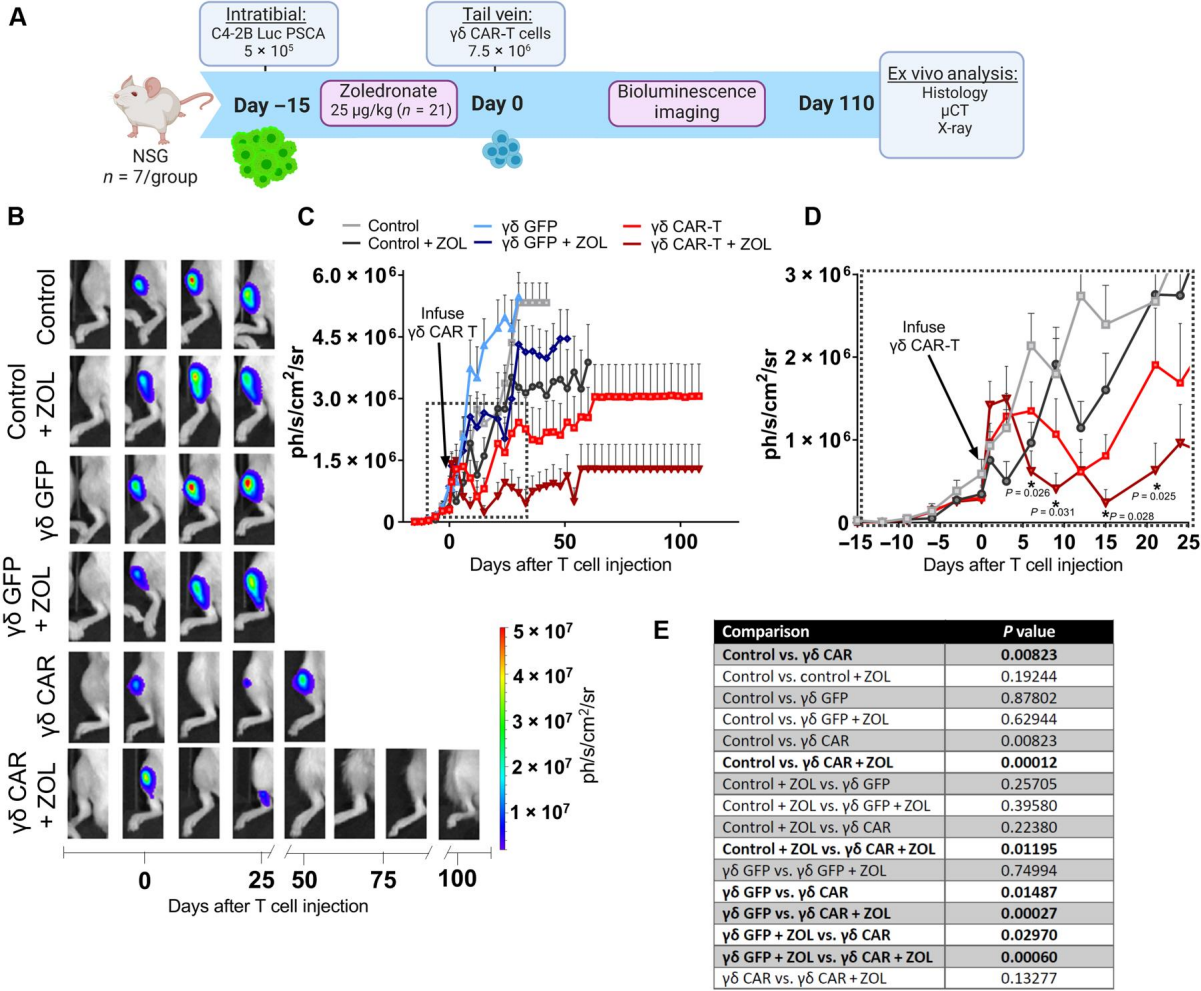
Enhancement of γδ-enriched CAR-T cell–mediated regression of intratibial prostate tumors by ZOL. (**A**) Schematic of in vivo experimental design to assess the antitumor efficacy of γδ CAR-T cells using an intratibial injection model of bone metastatic prostate cancer. (**B**) Representative bioluminescence images from control (untreated), γδ GFP, and γδ CAR-T–treated C4-2B PSCA^+^ tumor-bearing mice ± ZOL. (**C**) Quantification of tumor bioluminescence presented as mean luminescence (photons s^−1^ cm^−2^ sr^−1^) from control γδ GFP– and γδ CAR-T (± ZOL)–treated C4-2B PSCA^+^ tumor-bearing mice (*n* = 7 mice per group). γδ CAR-T cells were delivered on day 0 as indicated by an arrow. (**D**) Enlarged view of dashed inset from (C) highlighting rapid regression of tumors in γδ CAR-T– and γδ CAR-T + ZOL–treated mice relative to controls. Asterisks denote statistical significance between γδ CAR-T– and γδ CAR-T + ZOL–treated mice at specified imaging days. (**E**) Statistical comparisons from tumor growth curves presented in (C). Statistical analyses were performed by AUC calculation and two-sample *t* test between independent treatment groups, and significant findings are denoted with bold font.

On the basis of our in vitro data (Fig. 5), we hypothesized that ZOL would enhance the antitumor effects of γδ CAR-T in vivo. To this end, the left and right tibias of male NSG mice were intratibially inoculated with C4-2B PSCA^+^ and divided into two groups (*n* = 21 per group), with one cohort receiving vehicle [phosphate-buffered saline (PBS)] and the other receiving ZOL (25 μg/kg, every other day) (Fig. 6A). In contrast to our prior in vivo study (Fig. 3), tumors were allowed to establish for an extended period (2 weeks) to recapitulate a more aggressive disease state. Mice in the PBS and ZOL groups were further randomized, based on bioluminescence, into the six groups to be tested (Fig. 6B, day 0). Given the robust and persistent regression of established tumors observed in our prior in vivo study (Fig. 3), we reduced the dose of infused γδ CAR-T cells by 50% (7.5 × 10^6^ cells per injection) to better discern differences in antitumor effects between the vehicle control and ZOL-treated mice. Subsequent in vivo imaging revealed a rapid regression of tumor burden in all mice receiving γδ CAR-T cells within 7 days of γδ CAR-T cell infusion (Fig. 6, B and C). To test the duration of this response, tumor progression/regression was monitored for 110 days after γδ T cell transfer. Although γδ CAR-T cells alone provided remarkable and significant tumor regression in bone compared to untreated control (*P* = 0.00823) or to γδ GFP T cells (*P* = 0.01487), the combination of ZOL with γδ CAR-T cells produced a steeper response compared to γδ CAR-T cells alone, albeit not statistically significant (*P* = 0.13277). However, further examination of the initial regression rates found that ZOL yielded a more rapid effect in the γδ CAR-T cohort compared to γδ CAR-T alone (Fig. 6D) that was in agreement with our in vitro findings (Fig. 5). Analysis of serum levels of human prostate-specific antigen (PSA) from biweekly phlebotomy samples supported our bioluminescence observations demonstrating the effectiveness of γδ CAR-T and γδ CAR-T + ZOL compared to other groups (fig. S9, A and B). At study endpoint, we observed that ZOL also enhanced survival, with a median of 84 days for γδ CAR-T cell + ZOL versus 51 days for mice receiving γδ CAR-T cells + vehicle, but this difference was not statistically significant between those groups (figs. S9, C and D, and S10). Notably, ZOL treatment did yield a marked increase in the number of mice undergoing complete tumor regression following CAR-T treatment, with 60% of mice (*n* = 3 of 5) showing no luminescent signal or histologically detectable tumor at study endpoint versus 28.6% (*n* = 2 of 7) in the γδ CAR-T cell group without ZOL (fig. S10). Consistent with our earlier studies, γδ CAR-T cell therapy provided a significantly improved survival over control γδ T cells (*P* = 0.0206) (fig. S9D). Collectively, these findings indicate that ZOL enhances γδ-enriched CAR-T cell therapy–induced regression of prostate cancer in bone immediately after T cell transfer.

### γδ CAR-T cells accumulate in tumor-bearing bones following systemic infusion

**Fig. 7.**
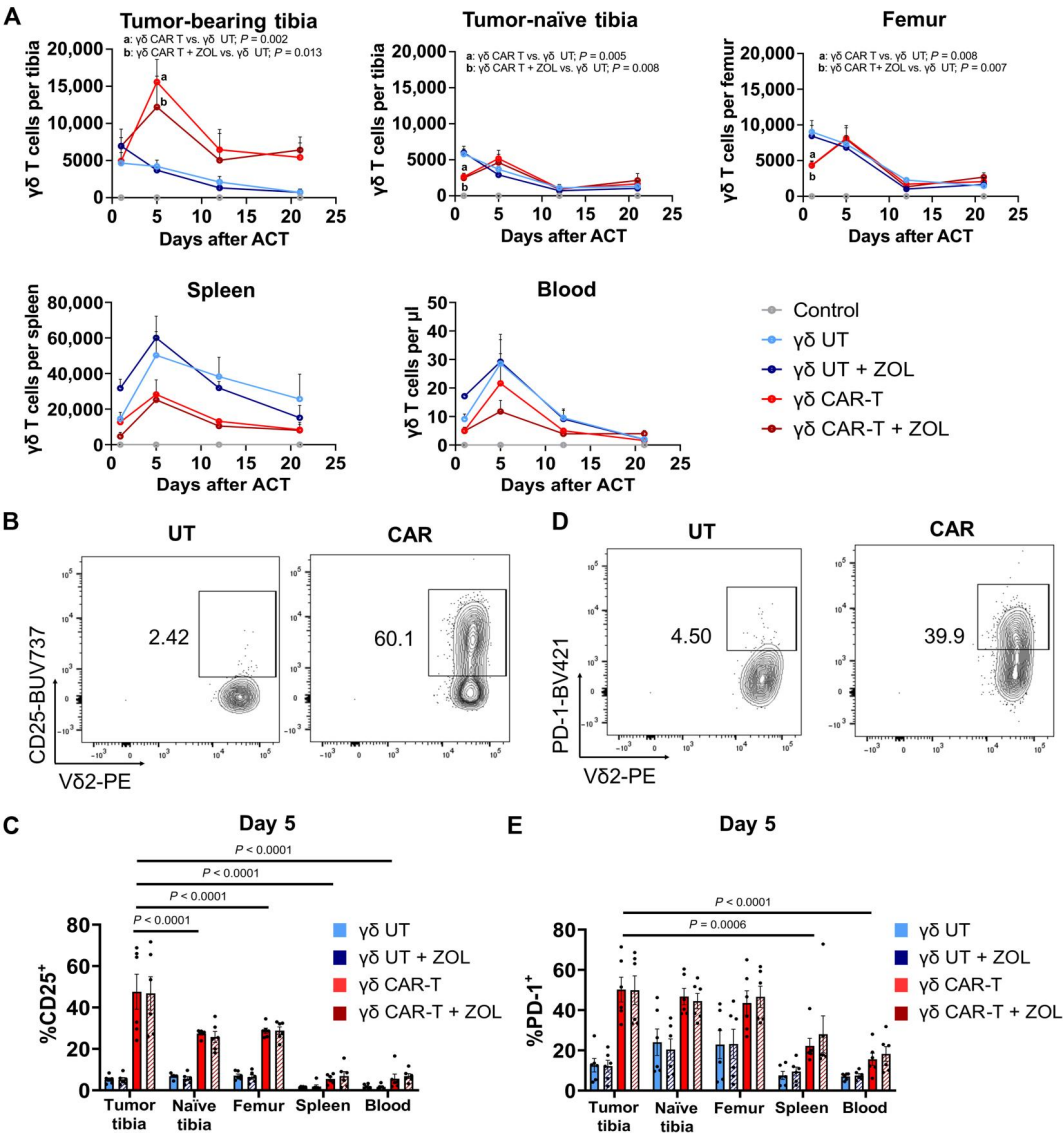
Accumulation of γδ CAR-T cells in tumor-bearing tibia. (**A**) Number of γδ T cells after adoptive cell therapy (ACT) in the different locations. Results depicted as means ± SEM of *n* = 3 to 6 mice per time point (except for untreated group *n* = 2 mice per time point) in two different experiments. Statistical analyses were performed by two-way ANOVA. Significance with respect to the UT group is shown. Only significant values are shown. (**B**) Representative dot plots of CD25 expression in γδ T cells at day 5 in mice from UT and CAR groups. Cells were gated on lymphoid, single, viable, CD45^+^, CD3^+^, Vδ2^+^. (**C**) Expression (%) of CD25 in γδ T cells at day 5 after ACT in the different locations. (**D**) Representative dot plots of PD-1 expression in γδ T cells at day 5 in mice from UT and CAR groups. Cells were gated on lymphoid, single, viable, CD45^+^, CD3^+^, Vδ2^+^. (**E**) Expression (%) of PD-1 in γδ T cells at day 5 after ACT in the different locations. Results depicted as means ± SEM of *n* = 6 mice per time point in two different experiments. Statistical analyses were performed by two-way ANOVA. Significance with respect to the CAR group in the different locations is shown.

Prompted by the observation that γδ CAR-T cells induce a rapid reduction in tumor burden following infusion, we decided to characterize their biodistribution during the first 3 weeks after transfer. Using the same experimental conditions (Fig. 6), 5.5 × 10^6^ anti-PSCA γδ CAR-T cells per mouse were injected into the recipient mice that were subsequently sacrificed at days 1, 5, 12, and 21 after infusion. The number of γδ T cells was then quantified in the bone marrow of tumor-bearing tibia, contralateral tumor-naïve tibia, tumor-naïve femur, spleen, and blood. Our data show that the number of γδ T cells increased after infusion, peaking at day 5, in the γδ CAR-T–treated groups (*P* = 0.002 in CAR-T versus UT-T, *P* = 0.013 in γδ CAR-T + ZOL versus UT-T), but not in the UT-T–treated groups (Fig. 7A). This effect was not observed in tumor-naïve bones or in spleen and blood, suggesting that it was not reflective of an overall overabundance of γδ CAR-T cells, but rather an active accumulation due to the presence of tumor cells. Treatment with ZOL did not affect the accumulation of γδ CAR-T cells.

We next analyzed the expression of CD25, as a marker of activation at the time of maximum accumulation (Fig. 7B). CD25 expression was significantly higher in γδ T cells recovered from CAR-T–treated tumor-bearing tibia than in any other tissue (*P* < 0.0001 for tumor-bearing versus tumor-naïve tibia, *P* < 0.0001 for tumor-bearing tibia versus femur, *P* < 0.0001 for tumor-bearing tibia versus spleen, and *P* < 0.0001 for tumor-bearing tibia versus blood). This pattern was observed only in mice injected with γδ CAR-T cells, irrespective of whether the mice had been pretreated with ZOL (Fig. 7C), and the overall CD25 expression was markedly reduced by day 21 (fig. S11A). Moreover, we also analyzed the expression of programmed cell death protein 1 (PD-1) as a marker of activation and of susceptibility to suppression by programmed death-ligand 1 (PD-L1) (Fig. 7D). PD-1 expression was also higher for the CAR-T–treated group in tumor-bearing tibia versus spleen (*P* < 0.0006) and versus blood (*P* < 0.0001), but no statistically significant differences were observed among the T cells that infiltrate the bone. Contrary to the pattern of CD25 expression, PD-1 levels increased from days 5 to 21, suggesting a less activated, more suppressed status (fig. S11B). PD-1 levels were also not affected by pretreatment with ZOL (see fig. S11C for complete statistical analysis of all groups).

Overall, these results indicate that the presence of tumor cells in the bone induces a CAR-dependent accumulation of T cells. This accumulation is associated with an activated phenotype that eventually declines by day 21. Furthermore, the enhancement in early tumor regression induced by ZOL does not affect PD-1 or CD25 expression, suggesting that combined anti-PD-1/PD-L1 agents could further improve responses for mCRPC.

### ZOL enhances γδ CAR-T–mediated protection against cancer-induced bone disease

**Fig. 8.**
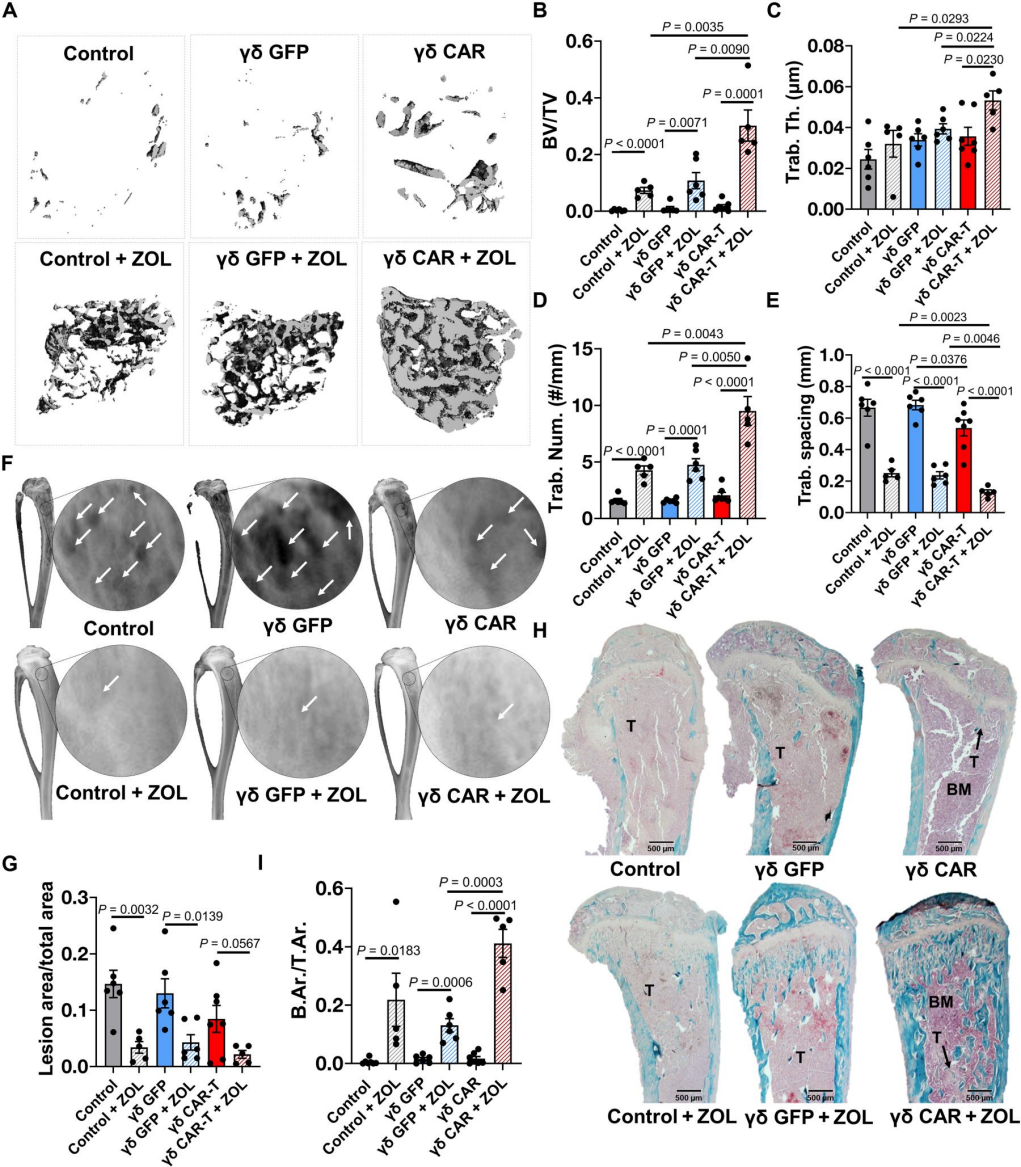
Enhancement of γδ-enriched CAR-T cell protection from tumor-induced osteolysis by ZOL. (**A**) Three-dimensional reconstructions of trabecular bone (top-down view) from high-resolution μCT scanning of tumor-naïve, control (untreated), and γδ CAR-T cell (± ZOL)–treated mice. (**B**) Trabecular bone volume, (**C**) trabecular thickness, (**D**) trabecular number, and (**E**) trabecular spacing measurements derived from μCT bone reconstructions. (**F**) Representative x-ray images illustrating osteolytic lesions (arrowheads) in tibias from tumor-naïve, untreated control, and γδ CAR-T cell (± ZOL)–treated mice. (**G**) Quantification of osteolytic lesion area from x-ray images, presented as ratio to total bone area. (**H**) Trichrome-stained tibia sections illustrating trabecular bone area (aquamarine), tumor (indicated with “T,” light pink), and bone marrow (indicated with “BM,” purple). (**I**) Quantification of trabecular bone area in trichrome-stained tissue sections. Trabecular area was measured using ImageJ and presented as ratio to total area. *n* = 5 to 7. Each dot represents a sample. Statistical analyses were performed by two-tailed *t* test, unpaired.

We next examined the impact of ZOL addition to γδ CAR-T cell infusion on cancer-induced bone disease. μCT analysis of tibias isolated from C4-2B PSCA^+^ intratibial studies (Fig. 6) showed that the addition of ZOL to γδ CAR-T cell therapy significantly protected bone volume and architecture in tumor-inoculated mice (Fig. 8, A to E). Notably, the γδ CAR-T + ZOL group had superior bone metrics compared to the γδ GFP + ZOL group, which was likely due to the targeted and rapid mCRPC debulking (Fig. 8, B to E). Imaging also demonstrated that treatment with ZOL significantly reduced tumor-induced osteolysis in control and γδ GFP T cell–treated groups (Fig. 8, F and G). A similar trend was observed in the γδ CAR-T group but did not reach statistical significance (*P* = 0.0567).

Analysis of sections derived for each group confirmed the enhanced bone-protective effects of γδ CAR-T cell + ZOL compared to either γδ GFP T cell + ZOL or control + ZOL (Fig. 8, H and I, and fig. S12). Consistent with bioluminescent imaging (Fig. 6, B and C), tumors were only detected in 71.4% (*n* = 5 of 7) of the γδ CAR-T and 40% (*n* = 2 of 5) of the γδ CAR-T cell + ZOL group. Together, our ex vivo examination of tumor-bearing bones indicates that the mitigation of cancer-induced bone disease by γδ-enriched CAR-T cell therapy is enhanced by ZOL.

## DISCUSSION

Despite advances in immunotherapy for other solid malignancies, there has been little success or momentum with respect to bone mCRPC apart from sipuleucel-T, an autologous dendritic cell vaccine that while U.S. Food and Drug Administration (FDA)–approved offers a modest 4-month increase in overall median survival (*33*, *34*). Here, we used an understudied subset of T cells (γδ T cells) as a vehicle to express a PSCA recognizing CAR and demonstrate that these cells promoted prostate cancer cytotoxicity in vitro and regression/eradication of established bone mCRPC cells in vivo. Excitingly, we also leveraged an FDA-approved bisphosphonate, ZOL, that is commonly prescribed to men with bone mCRPC to improve γδ CAR-T cell activity in vitro and in the bone-tumor microenvironment. This strategy offers a dual-targeting mechanism to eradicate bone metastatic prostate cancer, a strategy that can be quickly translated to the clinical setting.

Fundamentally, we show that Vγ9Vδ2 T cells provide a suitable platform for expression of CD28-costimulated second-generation CARs targeting PSCA, with transduction efficiencies on par with αβ T cells. When expressed in γδ T cells, our CAR mediated a robust cytokine release and antigen-specific cytotoxicity upon challenge with prostate tumor cells in vitro. Cytotoxicity was significantly increased when the tumor cells were pretreated with ZOL to induce the accumulation of phosphoantigens (Fig. 5). This crucial observation indicates that upon ex vivo expansion and gene modification, Vγ9Vδ2 T cells retain a functional TCR, which can be activated in concert with the CAR to maximize antitumor activity. This feature, unique to Vγ9Vδ2 T cells, may allow for the establishment of an “OR” gate without the need for coexpression of two synthetic immune receptors (*35*–*38*). Alternatively, if activation of the TCR is not potent enough to mediate tumor debulking, our data show that it can still enhance the effect of the CAR (Fig. 6).

Currently, the mechanism of activation of the Vγ9Vδ2 TCR has not been fully elucidated. Butyrophilin 3A1 (BTN3A1) and BTN2A1 are known to mediate the interaction between intracellular phosphoantigens in target and Vγ9Vδ2 T cells (*39*, *40*). In addition, γδ T cells may be activated via coordination of their NKG2D receptor and expression of ULBP1 and MICA/B stress ligands on target cells. Here, we show that these stress-associated ligands, in addition to BTN3A1, are expressed by bone metastatic prostate tumors (Fig. 1), and their expression in biopsies may help select patients who will respond best to γδ CAR-T cell therapies in prospective clinical trials. Additional studies will be required to quantify the contribution of NKG2D to the activation, costimulation, and/or cytotoxic potential of Vγ9/Vδ2 CAR-T cells.

The benefit of γδ T cells for use as CAR vehicles can be further attributed to their endogenous roles in first-line innate defense, lack of major histocompatibility complex (MHC) restriction (*41*), and natural tropism for the tumor microenvironment (*42*, *43*). Furthermore, the use of the Vγ9Vδ2 subtype is particularly attractive due to their predominance in peripheral blood and ability to be readily expanded to clinically relevant numbers with ZOL, which is already FDA-approved for the treatment of men with bone metastatic prostate cancer (*44*–*46*). A recent study of a 14-day ZOL-driven expansion protocol reports a 185-fold cellular expansion while maintaining purity of 99.5% γδ TCR^+^ cells (*47*). In addition, the antitumor activity of Vγ9Vδ2 T cells in preclinical studies and early phase clinical trials has illustrated an impressive safety profile for transfer of these cells that augurs well for their translation to the clinical setting (*48*).

Our work demonstrates that anti-PSCA γδ CAR-T cells potently promote the cytotoxicity of CRPC cell lines (C4-2B and 22Rv1) and that this effect could be enhanced via the addition of ZOL. Similar results were found in vivo, where the anti-PSCA γδ CAR-T induced regression and, in some mice, eradication of established tumors, especially in the ZOL treatment arm (60% with no evidence of disease at study endpoint). This effect was associated with increased degranulation and cytokine secretion, but not with an increase in PD-1 expression induced by ZOL. These tumor regressions are remarkable given the robustness and aggressiveness of the C4-2B model of CRPC in bone. However, some tumors did recur subsequent to anti-PSCA γδ CAR-T, raising the question of whether these recurrent prostate cancer cells retain PSCA expression and to what degree, or if a subpopulation of PSCA-negative cancer cells evolved. PSCA is expressed strongly in >99% of human bone metastatic tumor cells and minimally in other tissues, suggesting high potential efficacy of the approach in the clinical setting (*20*). In future studies, we will dissect this mechanism with experiments that include a second infusion of anti-PSCA γδ CAR-T cells to test rechallenging. These studies are presently being explored in clinic, where a second dose of CD19-targeting αβ CAR-T cells provided response in 39% of patients with B cell malignancies who did not experience durable response after the first infusion (*49*).

In addition to our bioluminescence measurements for tracking tumor burden, we also performed enzyme-linked immunosorbent assay (ELISA) to measure circulating PSA to include a clinically used measure of disease response. We noted a significant decline in PSA following treatment with γδ CAR-T cells but not with γδ GFP T cells. The addition of ZOL to γδ GFP T cells yielded a significant reduction in serum PSA, indicating presumable T cell activation via the endogenous γδ TCR due to accumulated IPP in target cells; however, it was still not as effective as γδ CAR-T cells without ZOL. This observation may reflect the fact that activation of the endogenous TCR alone does not result in a cytotoxic response that is strong enough to eradicate bulky tumors, which may explain why clinical trials based on transfer of unmodified γδ T cells plus ZOL have proven safe but largely ineffective (*50*). In contrast, γδ TCR activation in concert with CAR activation can enhance therapeutic efficacy. Note that no evidence of graft-versus-host disease was observed in mice, even several weeks after adoptive transfer. This has been reported for other forms of γδ T cell–based immunotherapy (*41*) and further strengthens the rationale for using purified γδ T cells in the allogeneic setting.

In our studies, γδ CAR-T cell administration was associated significantly with reduced cancer-induced bone disease assessed by μCT, x-ray, and histomorphometry, and this effect could be enhanced by combination with ZOL. This protection may be a consequence of anti-PSCA γδ CAR-T eradicating the cancer cells, but it is also possible that either the γδ CAR-T– or γδ CAR-T–derived factors subsequent to activation can have a direct effect on the bone stroma. The impacts of γδ CAR-T on the bone stroma are completely unknown at this juncture, but our data point to a protective effect. This is important since the extensive bone remodeling induced by prostate tumors substantially contributes to patient morbidity and anti-PSCA γδ CAR-T could mitigate this clinically significant issue.

Few previous studies have explored CAR-T cells for prostate cancer, and fewer still specifically address metastatic disease (*51*). However, some αβ T cell–based CAR-T cell products designed to target prostate-specific membrane antigen (PSMA) and PSCA generated within the past decade have reached clinical development, and the results of these studies are forthcoming (NCT02744287; NCT04249947; NCT03089203; NCT02744287; NCT03873805) (*51*, *52*). Early data from a study of five patients treated with anti-PSMA αβ CAR-T therapy, given with low-dose IL-2, show that two of five patients achieved partial responses (*52*). More recently, it was reported that administration of an anti-PSMA CAR armed with a transforming growth factor–β (TGF-β) dominant-negative receptor induced a reduction of serum PSA levels in 4 of 13 patients treated, one of whom unfortunately experienced fatal dose-limiting toxicity (*53*). These findings highlight the need for potent yet safe products to treat patients with this condition.

Solid tumors, and the bone microenvironment even more so, pose a serious challenge for CAR-T cell trafficking. It has been reported that PSCA-directed αβ CAR-T cells could home to bone metastases in mice (*51*). However, a nearly 10-fold higher T cell dose was necessary to obtain response in an intratibial model compared to subcutaneous models of the same prostate cancer cells. In contrast, Vγ9Vδ2 T cells offer a valuable opportunity for dual targeting and activation by stimulatory tumor cells and phosphoantigens, such as IPP, generated by nBPs such as ZOL that are commonly given to men with advanced prostate cancer (*16*). Here, we show the sensitivity of this approach, whereby ZOL improved γδ CAR-T cell in vitro cytotoxicity and antitumor responses as well as protection from cancer-induced bone disease in vivo. This is consistent with studies in other contexts. For instance, preclinical investigations of anti-CD19 γδ CAR-T cells combined with ZOL pretreatment in mice demonstrated an enhanced clearance of leukemic cells (*47*). The same study also reported that CD19-directed γδ CAR-T cells could target CD19 antigen-negative leukemia cells when primed with ZOL. This is in accord with reports showing that bisphosphonate (BP)–induced accumulation of IPP in in vivo models of human breast cancer promotes Vγ9Vδ2 cytotoxicity (*17*, *18*). Furthermore, a notable activation of γδ T cells in disease-free breast cancer patients following a single dose of BPs has also been observed (*54*). In our studies, we noted a significant increase in trabecular bone in the γδ CAR-T cells + ZOL group compared to other ZOL groups. γδ CAR-T cell–mediated tumor regression is likely responsible for the mitigation of cancer-induced bone disease, but whether activated γδ CAR-T cells have more direct influences on the bone stroma (*55*) has not been investigated thus far.

It has been proposed that the combination of ZOL and γδ T cells may also directly influence their trafficking to tumor sites (*18*, *56*). In our model, no ZOL-induced increase of γδ (CAR) T cell abundance was observed, suggesting that this effect may be model dependent. However, we did observe a significant accumulation of CAR-T cells in tumor-bearing bones, suggesting either antigen-driven expansion or retention, or a combination thereof. While infused CAR-T cells rapidly disappeared from the blood and most tissues, they remained present in tumor-bearing tibia for at least 21 days after infusion. During this time, they gradually acquired expression of the co-inhibitory receptor PD-1 while losing markers of activation. At endpoint, γδ (CAR) T cells were virtually undetectable, begging the question of whether this therapy would benefit from co-administration with anti-PD-1/PD-L1 antibodies, or repeated cell dosing.

To our knowledge, this study is the first to evaluate γδ T cells for expressing CAR and treating bone metastatic prostate cancer. The Vγ9Vδ2 subset is especially suitable for CAR expression based on its relative abundance in peripheral blood, the straightforward protocols for their ex vivo expansion (*45*), and their potential in vivo synergism with ZOL. Here, we have demonstrated that Vγ9Vδ2 CAR-T cells are highly effective in reducing bone mCRPC burden and in protecting against cancer-induced bone disease. Furthermore, our data demonstrate that the combination of γδ CAR-T cells with the BP ZOL enhances these responses and leads to dual activation as evidenced by increased cytotoxicity and cytokine secretion. These findings provide strong rationale for the translation of γδ CAR-T cells to the clinical setting for the treatment of men with bone mCRPC that have failed available lines of chemotherapy and/or hormonal therapy.

## MATERIALS AND METHODS

### Study design

The objective of this study was to determine the efficacy of γδ CAR-T cell therapy for the treatment of bone metastatic prostate cancer and how this can be further enhanced by BPs. We hypothesized that the application of BPs, such as ZOL, would activate the γδ TCR and increase the efficacy of the CAR-T therapy. Prostate cancer cell lines were used in vitro, and an intratibial prostate cancer injection protocol was used to generate tumors in both left and right tibias (dual injection model) in vivo. Sample sizes were determined on the basis of previous experiments and review of the literature. The number of biological (*n*) and technical replicates is indicated in the figure legends. Animals were randomized before administration of CAR-T cells based on mean luminescent signal from intratibial tumors. Exclusion criteria for in vivo studies included death from non–tumor-related cause or animals with tumors originating outside of the bone marrow compartment due to error during injection. Data analysis was performed in an unblinded manner.

### Cell lines and reagents

For cell culture, RPMI 1640 with GlutaMAX, Dulbecco’s modified Eagle’s medium (DMEM), PBS (pH 7.4), Antibiotic-Antimycotic 100X, and Ammonium-Chloride-Potassium (ACK) Lysing buffer were obtained from GIBCO. X-VIVO medium was obtained from Lonza. Zoledronic acid was purchased from Selleckchem (catalog no. S1314). Nontreated 6-well and 24-well tissue culture plates were obtained from Corning. E-Plate 96 PET was from ACEA Biosciences. Lipofectamine 2000, IFN-γ Mouse anti-Human, Biotin (clone B133.5), and IFN-γ Mouse anti-Human (clone 2G1) were purchased from Invitrogen (Thermo Fisher Scientific). Heat-inactivated fetal bovine serum (FBS) was purchased from Gemini Bio-Products. RetroNectin was obtained from Takara Bio. Lymphocyte separation medium (LSM) was from MP Biomedicals. Bovine serum albumin (BSA) was obtained from Sigma-Aldrich. Human serum AB was purchased from Gemini Bio-Products. Proleukin (IL-2) was ordered from Prometheus Laboratories. Biotin-Protein L was purchased from GenScript. Anti-human CD45-BV785 (clone HI30), anti-human CD3-BV711 (clone OKT3), anti-human CD3-AF700 (clone SK7), ultra-LEAF purified anti-human CD3 (clone OKT3), anti-human PD-1–BV421 (clone EH12.2H7), 4′,6-diamidino-2-phenylindole (DAPI) dye, anti-human CD27-BV605 (clone O323), anti-human CD122 (IL-2Rb)-PE (phycoerythrin)/Cy7 (clone TU27), and anti-human CD277 (BTN3)-PE (clone BT3.1) were bought from BioLegend. Streptavidin-PerCP (peridinin chlorophyll protein)/Cy5.5 and anti-human CD107a-FITC (fluorescein isothiocyanate) (clone eBioH4A3) were purchased from eBioscience (Thermo Fisher Scientific). Anti-human TCRVδ2-PE (clone 123R3), anti-human TCRγ/δ-APC (allophycocyanin) (clone REA591), and anti-human TCRα/β-APC (clone REA652) were obtained from Miltenyi Biotec. Anti-human TCRα/β-PE (clone T10B9.1A-31), anti-human TCRα/β-APC (clone T10B9.1A-31), anti-human CD25-BUV737 (clone 2A3), and GolgiStop (containing monensin) were purchased from BD Biosciences. LIVE/DEAD near-infrared (IR) dead cell stain kit was bought from Invitrogen (Thermo Fisher Scientific). 1-Step Ultra TMB-ELISA Substrate Solution and HRP-Conjugated Streptavidin were from Thermo Fisher Scientific. Androgen-independent C4-2B (CRL-3155) and 22Rv1 (CRL-2505) cell lines were purchased from the American Type Culture Collection. 22Rv1 was modified to stably express PSCA using lentiviral transduction (catalog no. RC209136L4, OriGene). C4-2B was modified to express PSCA using a retroviral vector encoding the codon-optimized cDNA for PSCA (Integrated DNA Technologies) as previously described (*57*). Deidentified Healthy Donor Buffy Coats were obtained from OneBlood (Florida Blood Services, FL) or Lifesouth Community Blood Centers. All cell lines were periodically mycoplasma-tested and authenticated by short tandem repeat (STR) (Labcorp, DNA Identification Testing Division).

### Bioinformatic analyses

Molecular and clinical data of 118 mCRPC tumor samples from the SU2C/PCF Dream Team were downloaded (25 July 2022) from cBioPortal (www.cbioportal.org/) (*24*). This analysis consists of RNA sequencing data from tumor samples biopsied from prostate (*n* = 3), bone (*n* = 29), lymph node (*n* = 50), liver (*n* = 17), and other grouped soft tissue sites (*n* = 19). Similarly, 498 primary tumor samples from TCGA-PRAD were downloaded (20 March 2020, gene annotation based on GENCODE v22) from the GDC (Genomic Data Commons) data portal (https://portal.gdc.cancer.gov/). The TCGA-PRAD gene expression data include the expression levels of 60,483 genes in primary PRAD (*n* = 498) and normal tissues (*n* = 52) (*58*). The normalized FPKM (fragments per kilobase of transcript per million mapped reads) expression values of 14 TCR gamma (TRGVs) genes and two BTN genes (*BTN2A1* and *BTN3A1*) were retrieved for the integrative analysis in metastatic and primary prostate cancer in this work. In addition, normalized FKPM expression values of stress ligands (*ULBP1*, *MICA*, and *MICB*) and the gamma delta CAR-T cell target (*PSCA*) levels were assessed in metastatic prostate cancer samples. Heatmaps of genes in metastatic samples and bar charts of expression in different metastatic sites were generated using Prism v.9.4.1. Primary sample heatmaps were plotted using R package ComplexHeatmap.

### Human prostate cancer sample immunostaining analyses

TMAs containing 1-mm core samples from castration-resistant metastatic prostate cancer from 45 patients were provided by C. Morrissey at the University of Washington. The microarray included samples with metastasis in bone, liver, lymph node, and lung collected as part of rapid autopsy protocol (table S1). The Institutional Review Board of the University of Washington approved this study (protocol no. 2341). All rapid autopsy tissues were collected from patients who signed written informed consent under the aegis of the Prostate Cancer Donor Program at the University of Washington.

Multiparametric Immunofluorescence staining of the TMA using the VECTRA system was performed by Moffitt’s AQUA laboratory. Formalin-fixed and paraffin-embedded tissue samples were immunostained using the AKOYA Biosciences OPAL 7-Color Automation IHC Kit (Waltham, MA) on the BOND RX autostainer (Leica Biosystems, Vista, CA). The OPAL 7-color kit uses tyramide signal amplification (TSA) conjugated to individual fluorophores to detect various targets within the multiplex assay. Sections were baked at 65°C for 1 hour and then transferred to the BOND RX (Leica Biosystems). All subsequent steps (e.g., deparaffinization and antigen retrieval) were performed using an automated OPAL IHC procedure (AKOYA). OPAL staining of each antigen occurred as follows: Slides were blocked with AKOYA blocking buffer for 10 min and then incubated with primary antibody, MICA/B (Abcam, Rb polyclonal, 1:100, dye 650), at room temperature (RT) for 60 min followed by OPAL horseradish peroxidase (HRP) polymer and one of the OPAL fluorophores during the final TSA step. Individual antibody complexes were stripped after each round of antigen detection. This was repeated five more times using the following antibodies: δ-TCR (Santa Cruz Biotechnology, H-41, 1:100, dye540), ULBP1 (Proteintech, Rb polyclonal, 1:6000, dye520), ε-CD3 (DALO, Rb polyclonal, 1:400, dye620), BTN3A1 (Millipore Sigma, Rb polyclonal, 1:50, dye570), and PCK (Abcam, [KRT/1877R], 1:150, dye690). After the final stripping step, DAPI counterstain was applied to the multiplexed slide and removed from BOND RX for coverslipping with ProLong Diamond Antifade Mountant (Thermo Fisher Scientific). Autofluorescence slides (negative control) were included, which use primary and secondary antibodies omitting the OPAL fluors and DAPI. All slides were imaged with the Vectra3 Automated Quantitative Pathology Imaging System. Visualization of hematoxylin and eosin (H&E) staining and multiplexed immunohistochemistry (IHC) was performed in Aperio ImageScope (Leica Biosystems) and inForm 2.4.8 Tissue Analysis software (PerkinElmer) for single marker visualization. γ-TCR staining did not pass quality control and was not considered for further analysis.

For analysis, data were imported to inForm Tissue Analysis software v.2.4 (Akoya, CA) to generate the spectral library and algorithms for image analysis. Several algorithms based on morphology and specific biomarker expression were created for each type of tissue present in the TMAs. The algorithms then were applied to batched images of similar tissue type. The resulting images, masks, and data were used for further analysis using proprietary image cytometry software (AKOYA).

Image data were analyzed using FCS Express 7 Plus Image Cytometry Version (De Novo Software). Component data generated from cell segmentation were imported to analyze expression of stress markers in tumor cells. PCK was selected as a marker for tumor cells. The threshold for PCK-positive staining was established on the basis of the staining intensity on negative samples. Expression of BTN3A1, ULBP1, and MICA/B was analyzed on the PCK-positive population. Only those samples with more than 100 PCK^+^ cells were used for further analysis.

For statistical analysis, to estimate effects of sample origins and BP on immune marker expression level, a linear mixed effects model (LMEM) was fitted for individual IHC marker through the restricted maximum likelihood estimation method after confirming no significant interaction effect between BP and sample origins in the saturated model. The lme4 R package was used to fit the LMEM. A detailed model specification is as follows

$$y_{ijk}=\alpha +\beta_{j}+\gamma_{k}+b_{i}+\varepsilon_{ijk}$$

In the model, α represents the overall average expression of an immune marker in bone, a reference sample origin, under no BP treatment. β*_j_* is the amount of mean change due to BP treatment. γ*_k_* is a difference in mean of marker level between bone (reference) and each of other sample origins. *b_i_* is the random effect of the *i*th tissue on the marker expression level. ε*_ijk_* represents a random measurement error, which is assumed to be normally distributed.

### Peripheral blood mononuclear cell and γδ T cell activation and expansion

Peripheral blood mononuclear cells (PBMCs) from buffy coats were obtained by performing a reported Ficoll-Paque density gradient protocol (*59*). Briefly, buffy coats were diluted in one volume of 1× PBS (pH 7.4). Then, each 30 ml of diluted buffy coat was carefully transferred to a 50-ml conical tube with 15 ml of LSM. Tubes were centrifuged at 2500 rpm at 20°C for 20 min with the slowest break setting. Next, the monolayer of PBMCs was transferred from each gradient to new tubes, washed with PBS, and centrifuged at 1500 rpm at 20°C for 5 min. Each pellet obtained was resuspended in 2 to 3 ml of ACK buffer and incubated for 10 min for elimination of red blood cells, per the manufacturer’s instructions (Roche). Cells were then counted. For γδ T cell culture, cells were resuspended in RPMI 1640 medium [supplemented with 5% FBS, antibiotic, and IL-2 (100 IU/ml)]. Expansion from PBMCs was performed as previously described (*18*). Briefly, a total of 48 × 10^6^ cells were plated in 24-well plates at a concentration of 1 × 10^6^ cells/ml with 4 μM ZOL (*18*, *29*). After 3 days, the medium was replaced with new complete RPMI 1640 medium with 4 μM ZOL. Cells were used for transduction on day 5. αβ T cell expansion from PBMCs was performed by seeding cells at a concentration of 1 × 10^6^ cells/ml with anti-CD3 (OKT3) antibody for 48 hours, in X-VIVO medium [supplemented with 5% human serum, antibiotic, and IL-2 (300 IU/ml)]. αβ T cells were used for transduction on day 2 (*26*).

### Viral production and γδ T cell transduction

RD114-pseudotyped retroviruses derived from pMSGV1-PSCA-8t-28z were generated by transient transfection as previously described (*25*, *26*, *60*). γδ T cell transduction was performed on day 5 after PBMC stimulation with ZOL and IL-2. Briefly, plates were coated with 1.5 ml of RetroNectin per well (20 μg/ml), for 2 hours at RT. Then, RetroNectin was removed and the wells were blocked with 2 ml of PBS + 2% BSA blocking buffer for 30 min at RT. After removal of the blocking buffer, wells were carefully washed with PBS. The viral supernatant was added into each well at a ratio of 1:1 with DMEM (supplemented with 10% FBS and antibiotic). Plates were then centrifuged for 2 hours at 2000*g* at 32°C. Immediately after, T cells were carefully plated in the wells at a concentration of 3.5 × 10^6^ cells in 4 ml per well, using RPMI 1640 medium supplemented for γδ T cells. Cells were then centrifuged at 1000 rpm for 10 min at 32°C to maximize T cell:virus interaction. The next day, a second transduction was performed after which cells were incubated at 37°C, 5% CO_2_. Transduction of αβ T cells was done similarly, but T cells were plated at a concentration of 2 × 10^6^ cells in 4 ml per well.

After transduction, γδ T cells were stained with Biotin-Protein L (1:300 dilution) for 20 min, followed by incubation with streptavidin-PerCP/Cy5.5 (1:200 dilution) for 30 min to detect CAR expression. Surface staining was performed at the same time as streptavidin incubation, using CD45-BV785 (1:200 dilution) and CD3-BV711 (1:100 dilution) to gate for T cells, TCRVδ2-PE (1:50 dilution) and TCRα/β-APC (1:50 dilution) to segregate αβ from γδ T cells, CD27-BV605 (1:20 dilution), and CD122 (IL-2Rβ)-PE/Cy7 (1:20 dilution). PBS with 2% BSA was used as the staining buffer. Staining of tumor cell lines with CD277 (BTN3)–PE (1:20 dilution) was performed similarly. DAPI or LIVE/DEAD near-IR (1:500 dilution) was used as viability dye. Flow cytometry was performed on a FACSCanto II or LSRII flow cytometer using FACSDiva software (BD Biosciences) at the Moffitt Cancer Center Flow Cytometry Core and further analyzed with FlowJo software (BD Biosciences).

### xCELLigence real-time cytotoxicity assay

The cytolytic capacity of γδ T cells was tested using an xCELLigence real-time cytotoxicity assay (RTCA) Multiple Plates instrument (ACEA Biosciences, San Diego, CA, USA). E-plates were calibrated with 50 μl of medium. Then, 1 × 10^4^ PSCA-expressing or unmodified C4-2B or 22Rv1 cells in 50 μl per well volume were added and allowed to adhere for 24 hours at 37°C. Next, UT or CAR-expressing γδ T cells were added at the corresponding ratio in 50 μl per well. Immediately afterward, 50 μl of growth medium or growth medium containing 16 μM ZOL (final concentration, 4 μM) was added to each well to bring the final volume to 200 μl. Cell index measurements (impedance per well) were recorded every 15 min during target cell growth and at least for 60 hours after addition of the T cells. Each condition was run in triplicate. Ratios always referred to total T cells. Before analyzing the data, using the RTCA software (V 2.0.0.1301. 2012 ACEA Biosciences Inc.), the cell index was normalized to the time point at which T cells were added to the tumor cell culture. Cytolysis curves and further statistical analyses [one-way analysis of variance (ANOVA) and multiple Tukey tests] were performed with GraphPad Prism (V 8.3.0).

### Cytokine quantification

Cytokine quantification was performed as a correlate for γδ T cell activity in the presence or absence of the target cells (*61*). To this end, cocultures were performed in U-bottom 96-well plates. A total of 1 × 10^5^ C4-2B PSCA^+^ or 22Rv1 PSCA^+^ tumor cells and the corresponding ratio of total γδ T cells or γδ CAR-T were plated in each coculture. These were also incubated with 4 μM ZOL. Tumor cells or γδ T cells with medium only were used as negative controls. The coculture was incubated overnight, and the supernatant was collected and frozen for future use in ELISA or ELLA assays. The ELISA was set up using the coating antibody (IFN-γ Monoclonal Antibody 2G1, Thermo Fisher Scientific, M700A), biotinylated detection antibody (IFN-γ Monoclonal Antibody B133.5 Biotin, Thermo Fisher Scientific, M701B), and HRP-Conjugated Streptavidin (Thermo Fisher Scientific, #N100). To measure IFN-γ, plates were incubated with 3,3′,5,5′-tetramethylbenzidine (Thermo Fisher Scientific, #34028) and 0.08 M H_2_SO_4_ and read at 450 and 550 nm. ELLA assays for granzyme B, IFN-γ, IL-2, and TNF-α were performed following the manufacturer’s protocol (Biotechne, ProteinSimple).

### CD107a expression in γδ T cells

Cocultures of γδ UT or γδ CAR-T cells and C4-2B PSCA^+^ cells were performed as described above for cytokine quantification, using a ratio of 1:1 tumor cells:γδ T cells. In this case, CD107a-FITC (2 μl per well) and GolgiStop (0.133 μl per well) were added to the cells for the last 15 hours. Cells were then stained with LIVE/DEAD near-IR. Surface staining was done using CD45-BV785 (1:200 dilution) and CD3-BV711 (1:100 dilution) to gate for T cells and TCR γ/δ-APC (1:50 dilution) and TCRα/β-PE (1:100 dilution) to segregate αβ from γδ T cells. Flow cytometry was performed on an LSR II flow cytometer (BD Biosciences).

### Animal care

All animal experiments were performed under University of South Florida Institutional Animal Care and Use Committee (IACUC) approval (R1762; R7429) and in accordance with the *Guidelines for the Care and Use of Laboratory Animals* manual published by the National Institutes of Health. NSG mice were purchased from The Jackson Laboratory (#005557; Bar Harbor, ME). Animals were maintained on a 12-hour light/dark schedule and fed ad libitum.

### In vivo mouse models

C4-2B prostate cancer cells expressing luciferase and PSCA were grown to near confluency in complete medium (DMEM/F-12, 10% FBS, 1% penicillin/streptomycin) at 37°C, 5% CO_2_. Cells were trypsinized, washed three times with 1× PBS, and filtered using a 40-μm nylon filter. Cells were then counted and reconstituted for intratibial injection in PBS (20-μl volume per tibia) as we have described previously (*62*, *63*). Six-week-old male NSG mice were intratibially inoculated with 2 × 10^5^ or 5 × 10^5^ cells per tibia, depending on study. Subsequently, mice were imaged for bioluminescence every 2 days as a correlate of tumor growth (IVIS 200, Perkin Elmer). Once tumors had established (~10 days), mice were randomized and separated into cohorts. UT γδ T cells and anti-PSCA γδ CAR-T cells (1.5 × 10^7^, 7.5 × 10^6^, or 5.5 × 10^6^ cells per mouse, depending on study) were then administered via tail vein. Mice received 100,000 IU of IL-2 by intraperitoneal injection every 2 days. For studies with ZOL, mice were treated every other day, receiving 25 μg/kg by subcutaneous injection immediately following intratibial injection of cancer cells. ZOL was discontinued 1 day before administering γδ CAR-T cells (see schedule; Figs. 3A and 6A). At endpoint (1 × 10^6^ RLU average or days 1, 5, 12, 21, and 60, depending on study), hindlimbs were collected following euthanasia and fixed for 24 hours in 10% neutral-buffered formalin, or bone marrow was isolated via centrifugation (*64*), depending on the experiment. Spleen and blood were also collected on days 1, 5, 12, and 21. To analyze the number and phenotype of γδ T cells, cells were stained with LIVE/DEAD near-IR and Biotin-Protein L followed by incubation with Streptavidin-PerCP/Cy5.5 as described above. Surface staining was performed at the same time as streptavidin incubation, using CD45-BV785 (1:200 dilution) and CD3-AF700 (1:125 dilution), to gate for T cells, TCRVδ2-PE (1:50 dilution) and TCRα/β-APC (1:100 dilution) to segregate αβ from γδ T cells, CD25-BUV737 (1:100 dilution), and PD-1–BV421 (1:100 dilution). To quantify the number of γδ T cells at each time point, CountBright Absolute Counting beads (Invitrogen) were added before performing flow cytometry. Flow cytometry was performed on an LSR II flow cytometer.

### Ex vivo bone morphometry and histological analysis

Upon removal from the study, mouse tibias were isolated and prepared for histology by fixation in 10% neutral-buffered formalin for 24 hours. Radiographic images of tibias were acquired by x-ray using an energy of 70 kVp and an exposure time of 200 ms (Faxitron) and analyzed by ImageJ. Briefly, the sum of areas of bone resorption was quantified and calculated as a function of total bone area. For μCT analysis, the proximal tibia metaphyses were scanned (μCT-40; Scanco Medical). An evaluation of trabecular bone structural parameters (trabecular volume, trabecular thickness, trabecular number, and trabecular spacing) was performed within a 1-mm section beginning 500-μm distal from the growth plate. A three-dimensional cubical voxel model of bone was constructed, and calculations were made for relative bone volume per total volume and trabecular number (*63*, *65*).

After ex vivo x-ray and μCT analyses, bone tissues were decalcified in 14% EDTA, pH 7.4, for 21 days. Sections (5 μm) were stained using Gomori trichrome solution and counterstained with hematoxylin to visualize gross anatomy including tumor burden, bone marrow, and cortical and trabecular bone. Mice were removed from all final analyses if tumors were observed to be growing outside of the cortical bone. Trabecular bone area was measured from trichrome-stained sections by measuring the area of multiple sections (*n* > 3) per mouse in a 1-mm-long region beginning 0.5 mm from the growth plate. For all histological analyses, a minimum of three representative micrographs per section were acquired. Micrographs were analyzed and measured using ImageJ.

### Statistical methods

To determine statistical significance, sample sizes were greater than or equal to *n* = 3. *T* test or ANOVA followed by Tukey’s multiple or Holm-Šídák comparison test was performed. A *P* value of ≤0.05 was considered to be statistically significant. Data are presented as SEM. All statistical analyses were performed with GraphPad Prism 9.1.1 (GraphPad Inc., La Jolla, CA). Further analysis of the data was performed by the Moffitt Biostatistics and Bioinformatics Shared Resource. Competing risk analysis was performed to analyze survival outcomes. Cumulative incidence function of death analysis was estimated using the R cmprsk package and then compared using Gray’s test to analyze survival outcomes (*66*). This analysis considers competing risks independently of tumor-caused death. To assess tumor growth curves, the AUC was compared using a two-sample *t* test between independent treatment groups (*67*).

## Notes

View/request a protocol for this paper from *Bio-protocol*.
