# Supplementary material for: γδ-Enriched CAR-T cell therapy for bone metastatic castrate-resistant prostate cancer
Source: Sci Adv. 2023 May 3;9(18):eadf0108. doi: 10.1126/sciadv.adf0108 (PMC10156127; doi:10.1126/sciadv.adf0108)
Supplement: Supplementary file 1 — Supplementary Materials and Methods Figs. S1 to S12 Table S1 [file sciadv.adf0108_sm.pdf]

Science Advances
AAAS

Supplementary Materials for

γδ-Enriched CAR-T cell therapy for bone metastatic castrate-resistant prostate cancer

Jeremy S. Frieling *et al.*

Corresponding author: Jeremy S. Frieling, jeremy.frieling@moffitt.org;
Leticia Tordesillas, leticia.tordesillas@moffitt.org; Conor C. Lynch, conor.lynch@moffitt.org;
Daniel Abate-Daga, daniel.abatedaga@moffitt.org

Sci. Adv. **9**, eadf0108 (2023)
DOI: 10.1126/sciadv.adf0108

This PDF file includes:

Supplementary Materials and Methods
Figs. S1 to S12
Table S1

MATERIALS AND METHODS

Retroviral titration

Viral titers (expressed as viral particles per milliliter, VP/mL) were determined in retroviral supernatants with the use of the QuickTiter Retrovirus Quantitation Kit (Cell Biolabs, San Diego, CA), according to manufacturer instructions. Briefly, 2 mL of supernatant was collected, incubated at 37°C for 30 minutes with 10 µL of QuickTiter Solution A, supplemented with 20 µL of QuickTiter Solutions B1 and B2, incubated at 37°C for 30 minutes, and finally resuspended in 20 µL of QuickTiter Solution C before allocation to duplicate wells of a black plate with CyQuant GR Dye for data acquisition. Number of viral particles was calculated based on absorbance readings from a Synergy H1 Microplate Reader (BioTek, Winooski, VT) with a 480/520 nm filter set, using 1:2 serial dilutions of QuickTiter RNA Standard to produce a standard curve.

Quantification of CAR transgene copy number

For determination of the copy numbers of CAR transgene in PSCA-8t28z, PSCA-8t27z, PSCA-8tBBz, and PSCA-28t28z CAR-transduced gamma-delta T cells, DNA was extracted from transduced and untransduced T cells using the DNeasy Blood and Tissue Kit (QIAGEN, Venlo, Netherlands) according to manufacturer instructions. Standard curves were generated using serial dilutions of MSGV1-PSCA-8t28z and pET30-2-GAPDH (Addgene, #83910) plasmids to yield standard curves with 10^8, 10^7, 10^6, 10^5, 10^4, 10^3, 10^2, and 10 copies each. Extracted DNA was diluted with ddH_2O to yield 50 ng/well, then all samples were allocated into duplicate wells with TaqMan Universal PCR Master Mix (Applied Biosystems, 4304437) and TaqMan primer/probes sets specific for PSCA-CARs (Applied Biosystems, custom designed, described in (*25*) or GAPDH (Applied Biosystems, Hs02786624_g1). An absolute quantification qPCR program was run on an Applied Biosystems Real-Time PCR Instrument with default cycles. Results were expressed as copies of transgene per copy of GAPDH.

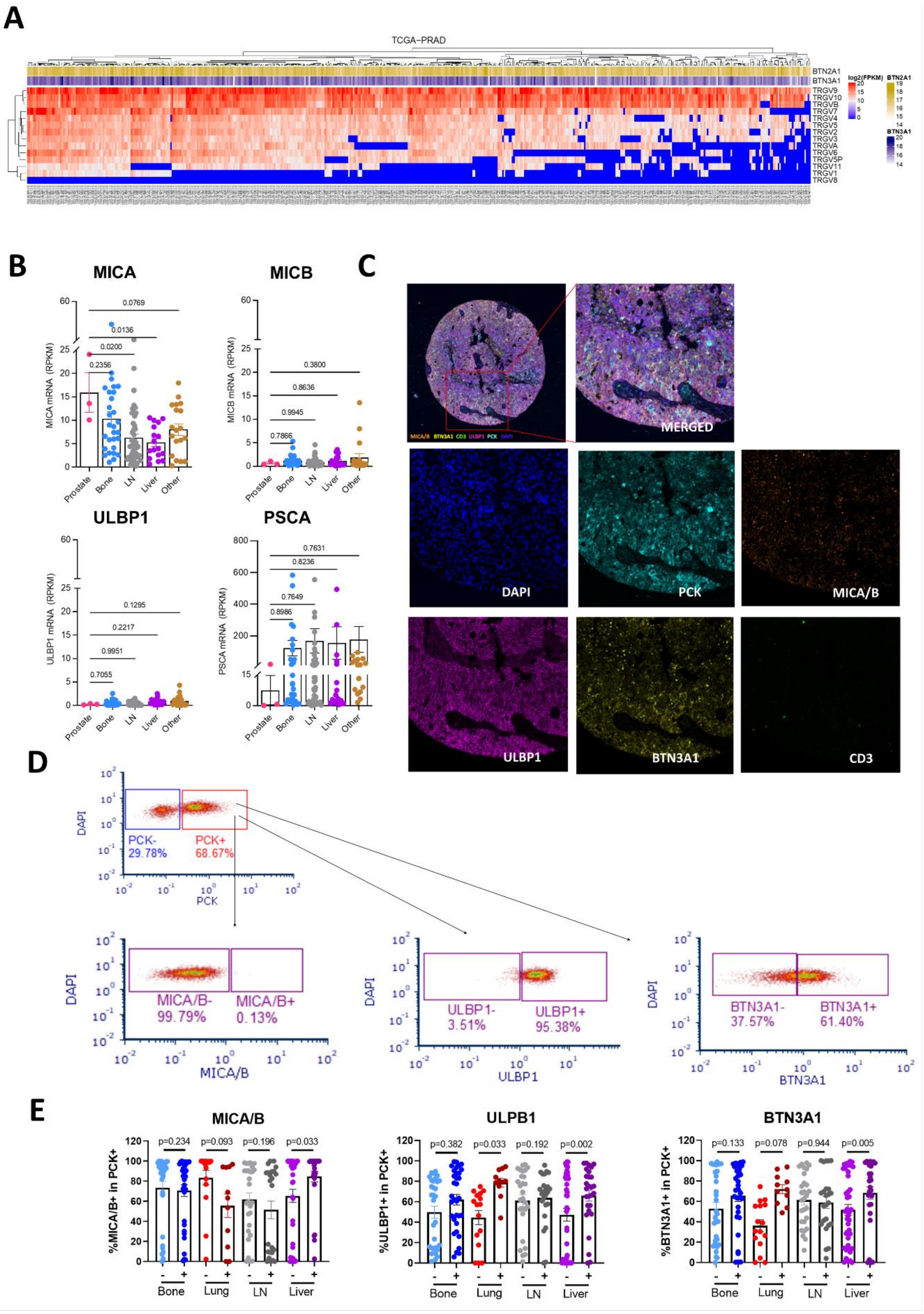
A
TCGA-PRAD
BTN2A1
BTN3A1
TRGV9
TRGV10
TRGVB
TRGV7
TRGV4
TRGV5
TRGV2
TRGV3
TRGVA
TRGV6
TRGV5P
TRGV11
TRGV1
TRGV8
log2(FPKM)
BTN2A1
BTN3A1
B
MICA
MICB
ULBP1
PSCA
MICA mRNA (RPKM)
MICB mRNA (RPKM)
ULBP1 mRNA (RPKM)
PSCA mRNA (RPKM)
0.0769
0.0136
0.0200
0.2356
0.3800
0.8636
0.9945
0.7866
0.1295
0.2217
0.9951
0.7055
0.7631
0.8236
0.7649
0.8986
Prostate
Bone
LN
Liver
Other
C
MICA/B BTN3A1 CD3 ULBP1 PCK DAPI
MERGED
DAPI
PCK
MICA/B
ULBP1
BTN3A1
CD3
D
DAPI
PCK
PCK-
29.78%
PCK+
68.67%
MICA/B-
99.79%
MICA/B+
0.13%
MICA/B
ULBP1-
3.51%
ULBP1+
95.38%
ULBP1
BTN3A1-
37.57%
BTN3A1+
61.40%
BTN3A1
E
MICA/B
ULPB1
BTN3A1
%MICA/B+ in PCK+
%ULBP1+ in PCK+
%BTN3A1+ in PCK+
p=0.234
p=0.093
p=0.196
p=0.033
p=0.382
p=0.033
p=0.192
p=0.002
p=0.133
p=0.078
p=0.944
p=0.005
Bone
Lung
LN
Liver

Fig. S1. TRGV, stress markers MICA/B and ULBP1, and PSCA expression in metastatic prostate tumors. (**A**) Bioinformatic analysis of *BTN3A1*, *BTN2A1*, and TCR gamma variable regions (*TRGV*) in 498 primary prostate tumors (TCGA-PRAD). (**B**) *MICA*, *MICB*, *ULBP1*, and *PSCA* expression in primary prostate tumors compared to bone, liver, lymph node, and other soft tissue metastasis tumor site from 118 individuals with mCRPC (SU2C/PCF). (**C**) Representative image of multiplex immunofluorescent staining of lymph node metastasis sample from tumor microarray (TMA) containing 1 mm cores. (**D**) Gating strategy followed for image analysis. (**E**) Percentage of the different markers in pan-cytokeratin (PCK) positive cells in metastatic samples from patients treated with bisphosphonate therapy (+) or non-treated (-). LN, lymph node. (n=64, 27, 52, 70 for bone, lung, lymph node and liver samples, respectively). Each dot represents a sample. Bars represent mean ± SEM. Linear mixed effect model was used for statistical analysis.

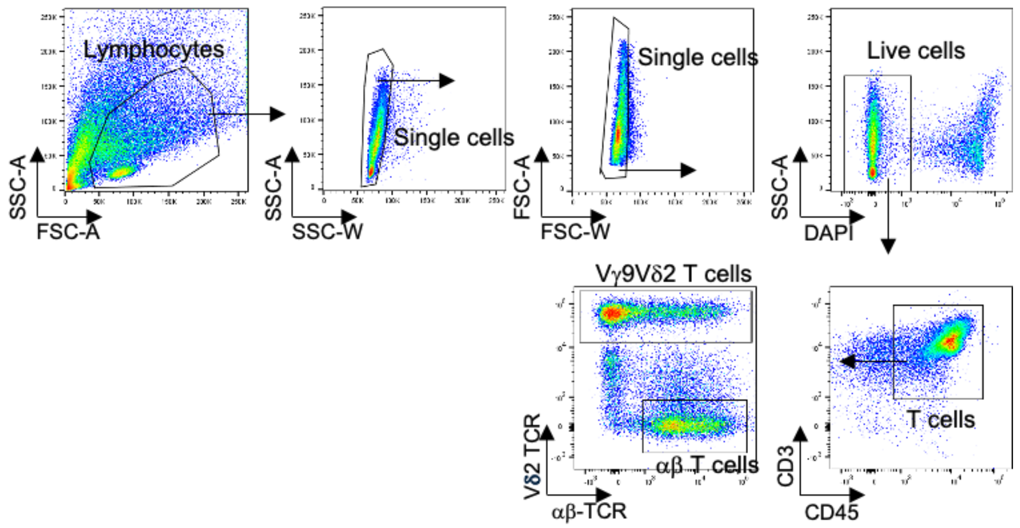

Fig. S2. Gating Strategy for γδ CAR-T cells. Gating strategy followed for flow cytometry in Figure 2 is described.

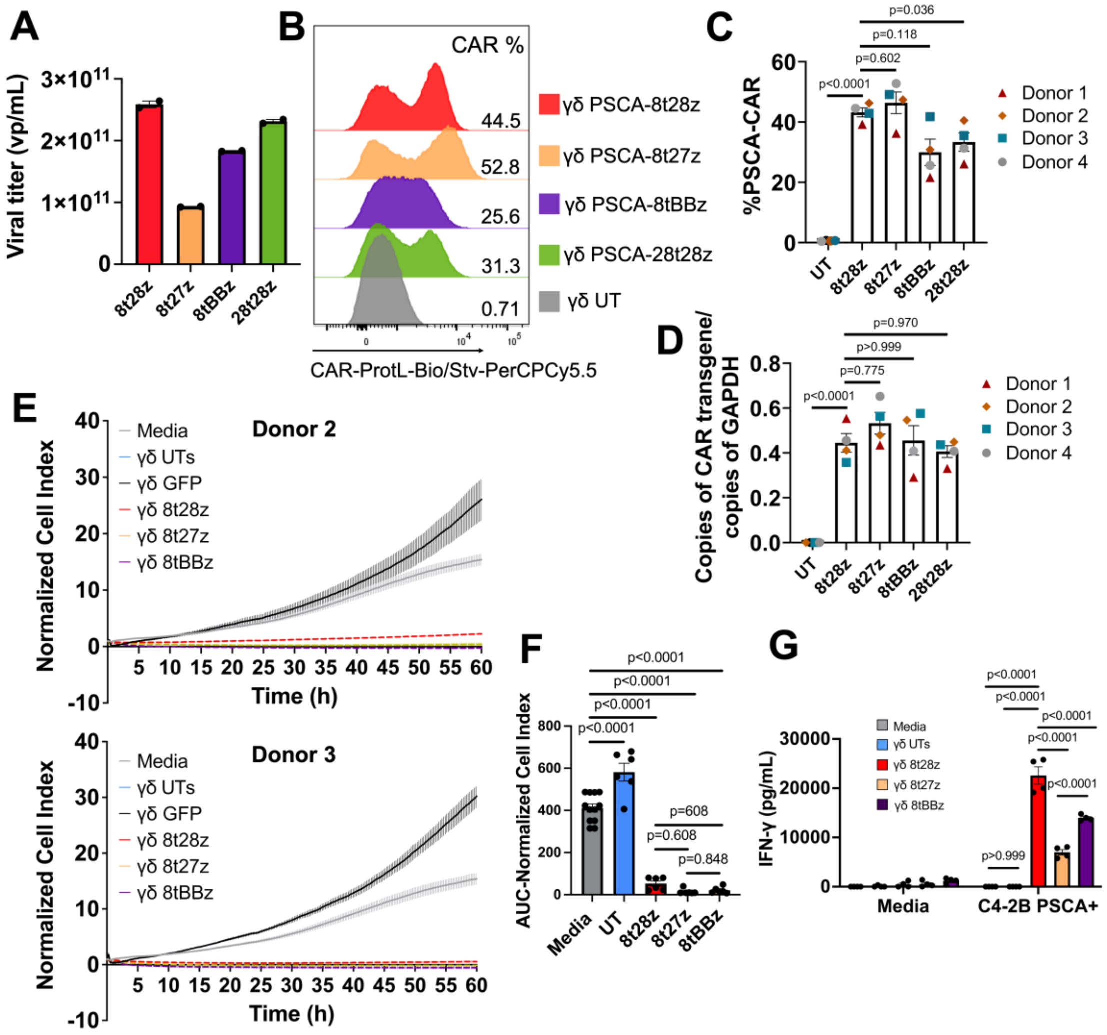

Fig. S3. Comparison of CAR designs. (**A**) Virus titration is shown as viral particles (vp)/mL for the different PSCA constructs. (**B**) Representative histogram of CAR expression for the different PSCA constructs in γδ T cells from the same donor. Gated on lymphoid, and single live $CD45^{+}CD3^{+}V\delta2^{+}$ cells. (**C**) CAR expression (%) for the different PSCA constructs in γδ T cells from 4 different donors. Statistically significant differences with respect to 8t28z are shown. Each dot represents a different donor. Statistical analyses performed by one-way ANOVA for matching samples, Dunnett's multiple comparisons test. (**D**) Vector copy number of each PSCA construct expressed as copies of transgene per copy of GAPDH. Statistically significant differences with respect to 8t28z are shown. n=4. Each dot represents a different donor. Statistical analyses

performed by one-way ANOVA for matching samples, Dunnett's multiple comparisons test. (**E**) Real time cytotoxic assay (RTCA) analysis with 2 different donors of C4-2B PSCA+ cells co-cultured in absence of T cells (Media), with UT γδ T cells or γδ CAR-T cells (ratio 1:2 Tumor: T cells), displaying different costimulatory domains (CD27, CD28, and 4-1BB) and the same transmembrane domain (CD8). (**F**) Quantification of AUC (Area Under the Curve) of the RTCA analysis shown in (E). Graph shows the average of the 2 donors. (**G**) Quantification of IFN-γ (ELISA) in supernatants from different γδ CAR-T cells co-cultured with C4-2B PSCA+ cells overnight. Graph shows average of 2 different donors. Percentage of CAR+ cells was normalized across samples. Bars represent mean ± SEM. Each dot represents a technical replicate. Statistical analyses performed by one-way ANOVA with multiple comparisons.

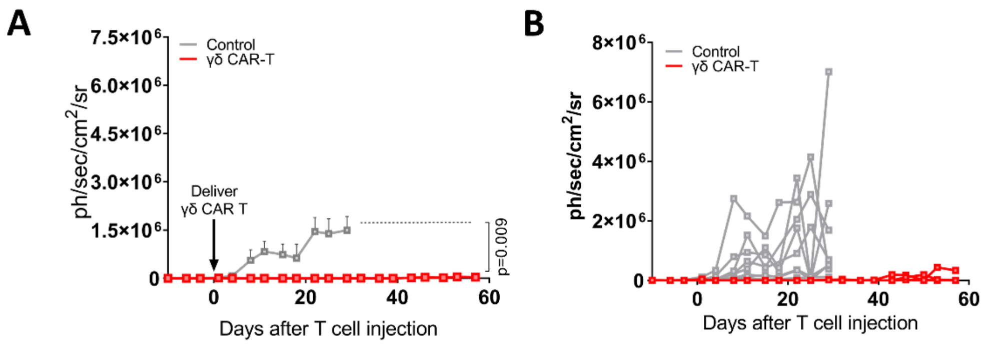

Fig. S4. Anti-PSCA γδ CAR-T cells mitigate prostate tumor growth in bone. (**A**) Mean luminescence of C4-2B PSCA+ intratibial tumors plotted with solid Y-axis. (**B**) Individual luminescence growth plots of left and right intratibial tumors over time. n=5 mice/group. Arrow indicates that γδ CAR-T cells were infused on Day 0.

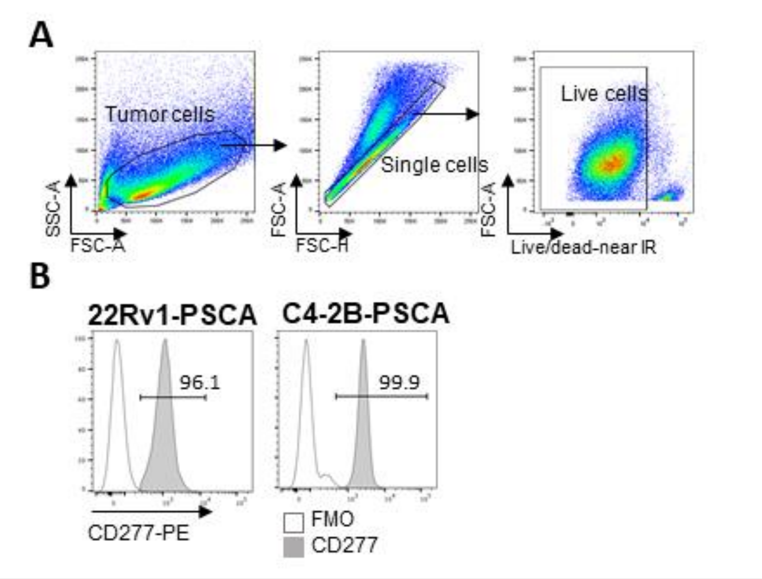

Fig. S5. BTN3 is expressed by metastatic prostate cell lines. (**A**) Gating strategy. (**B**) Representative histogram of CD277 (BTN3) expression in prostate cancer cell lines expressing PSCA analyzed by flow cytometry. Gates indicate percentage of live cells expressing CD277. FMO, Fluorescence Minus One. n=2.

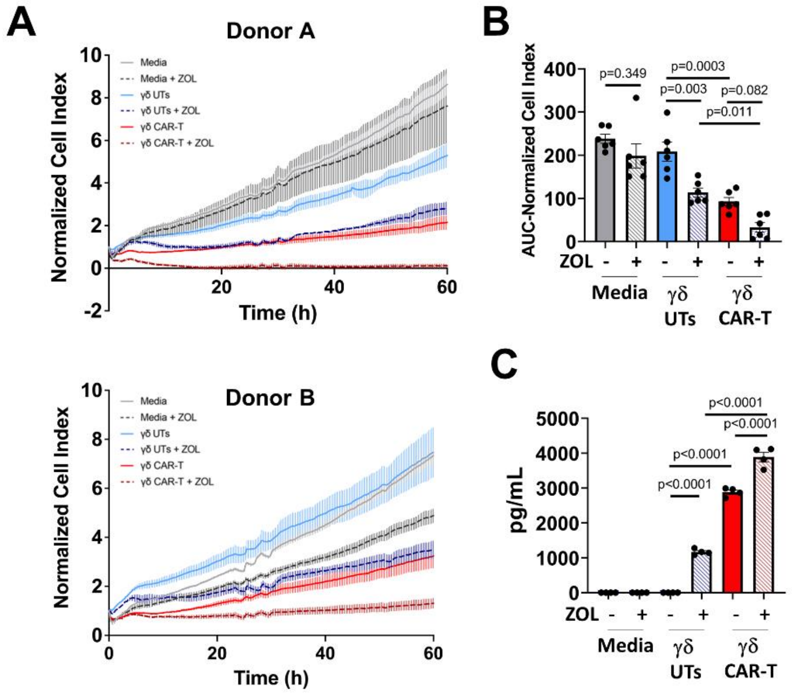

Fig. S6. Effects of ZOL on γδ CAR-T cell cytotoxicity against 22Rv1 PSCA+. (**A**) RTCA analysis of 22Rv1 PSCA+ cells with 2 different donors, co-cultured with γδ UT cells or anti-PSCA γδ CAR-T cells (ratio 1:1 Tumor: T cells) in the presence or absence of 4 μM ZOL. (**B**) Quantification of AUC (Area Under the Curve) of the RTCA analysis shown in (A). Graph represents average of the 2 donors. (**C**) IFN-γ release (ELISA) upon co-culture of 22Rv1 PSCA+ cells with media or the indicated T cell effectors (ratio 1:1 Tumor:T cells), in presence of 4 μM ZOL. Graph represents average of the 2 donors. Bars are shown as mean ± SEM. Each dot represents a technical replicate. Statistical analyses performed by one-way ANOVA, Holm-Šídák test.

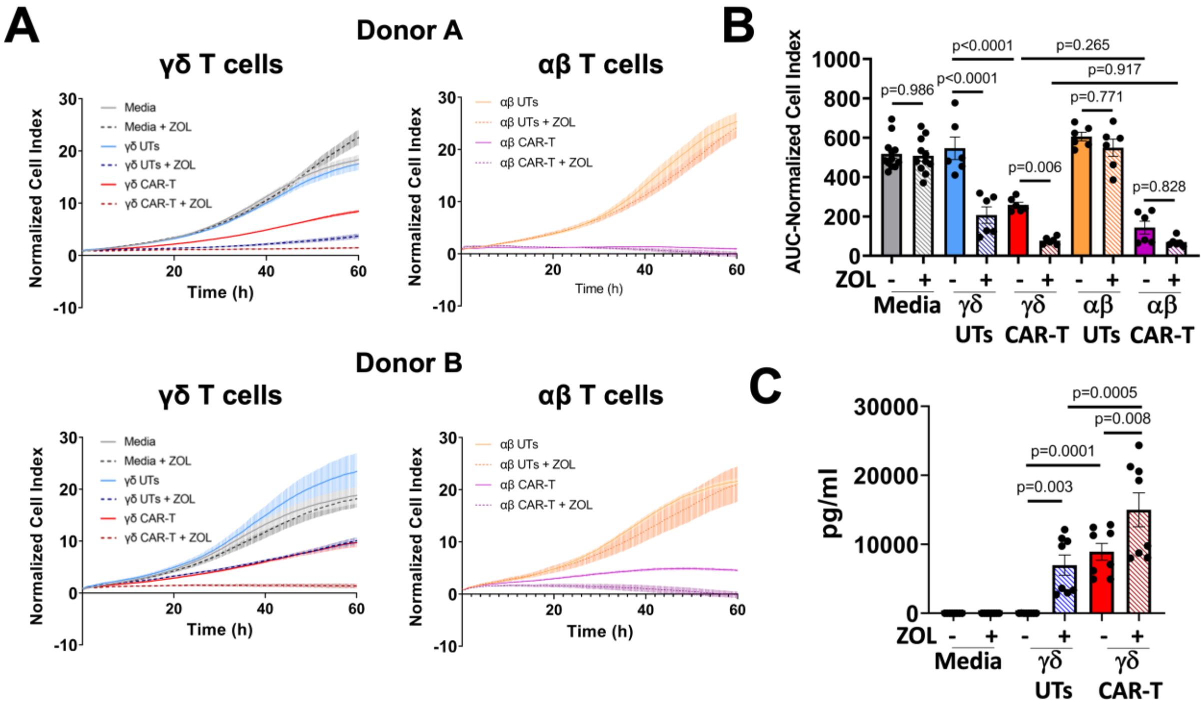

Fig. S7. Effects of ZOL on γδ CAR-T cell activity against C4-2B PSCA+. (**A**) RTCA analysis of C4-2B PSCA+ cells with 2 different donors, co-cultured with γδ UT cells and anti-PSCA γδ CAR-T cells (left) or with αβ UT cells and anti-PSCA αβ CAR-T (right) (ratio 1:0.5 Tumor: T cells) in the presence or absence of 4 µM ZOL. (**B**) Quantification of AUC (Area Under the Curve) of the RTCA analysis shown in (A). Graph represents average of the 2 donors. (**C**) IFN-γ release (ELISA) upon co-culture of C4-2B PSCA+ cells with media or the indicated T cell effectors (ratio 1:0.5 Tumor:T cells), in presence of 4 µM ZOL. Graph represents average of 2 donors. Bars are shown as mean ± SEM. Each dot represents a technical replicate. Statistical analyses performed by one-way ANOVA, Holm-Šídák test.

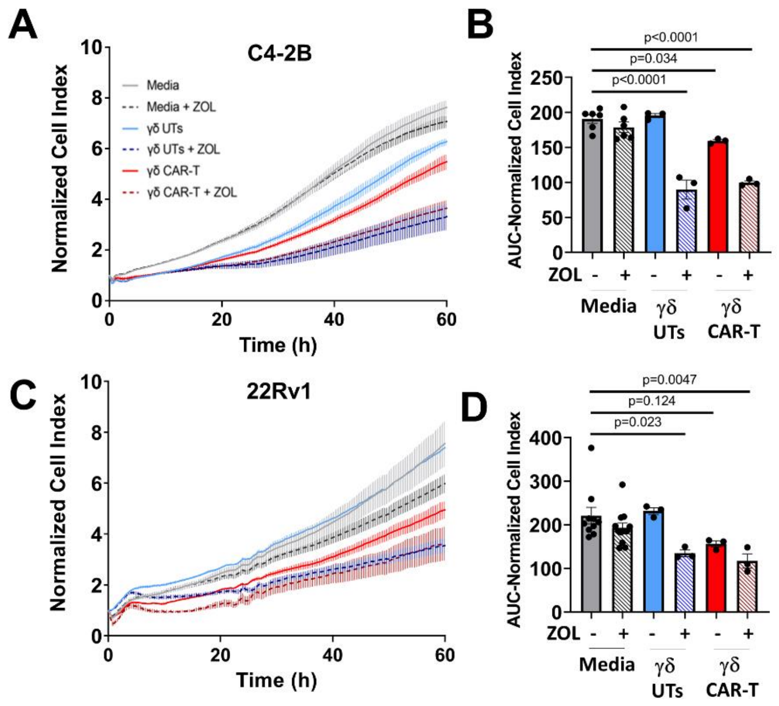

Figure S8. TCR-mediated recognition of PSCA-negative targets by γδ CAR-T cells. (**A**) RTCA analysis of unmodified C4-2B co-cultured with γδ UT cells or anti-PSCA γδ CAR-T cells (ratio 1:1 Tumor: T cells) in the presence or absence of 4 μM ZOL. (**B**) Quantification of AUC (Area Under the Curve) of the RTCA analysis shown in (A). (**C**) RTCA analysis of unmodified 22Rv1 co-cultured with γδ UT cells or anti-PSCA γδ CAR-T cells (ratio 1:1 Tumor: T cells) in the presence or absence of 4 μM ZOL. (**D**) Quantification of AUC (Area Under the Curve) of the RTCA analysis shown in (C). A representative donor is shown. Bars are shown as mean ± SEM. Each dot represents a technical replicate. Statistical analyses performed by one-way ANOVA, Dunnett's multiple comparison test.

A

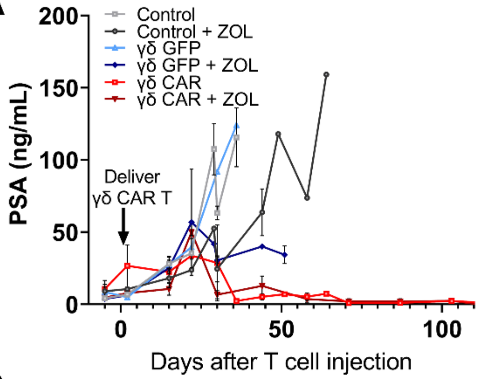

C

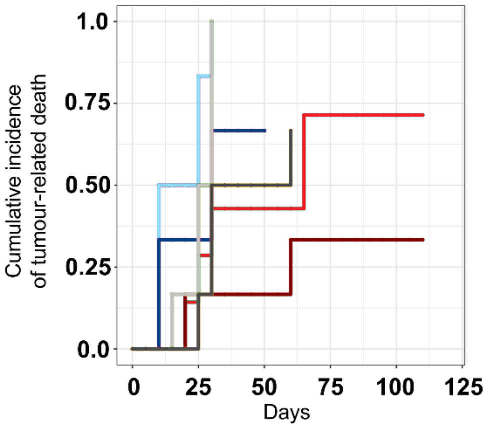

B

Comparison	*p-value*
Control vs. γδ GFP	0.3083
Control vs. γδ CAR	<0.0001
γδ CAR vs. γδ CAR+ZOL	0.3532
Control vs. Control+ZOL	0.016
γδ GFP vs. γδ GFP+ZOL	0.001
Control+ZOL vs. γδ GFP+ZOL	0.0063
Control+ZOL vs. γδ CAR+ZOL	<0.0001
γδ GFP vs. γδ CAR	<0.0001
γδ GFP+ZOL vs. γδ CAR+ZOL	<0.0001

D

Comparison	*p-value*
Control vs. Control+ZOL	0.0103
Control vs. γδ CAR+ZOL	0.0496
Control+ZOL vs. γδ GFP	0.0091
γδ GFP vs. γδ CAR	0.0206
γδ GFP vs. γδ CAR+ZOL	0.0025

Fig. S9. Detailed analyses of γδ-enriched CAR-T cell mediated regression of intratibial prostate tumors in combination with zoledronate. (**A**) Human PSA serum levels from bi-weekly serial collections from C4-2B PSCA+ tumor-bearing mice. Arrow indicates time at which γδ CAR-T cells were delivered (Day 0). (**B**) Table of statistical comparisons from PSA ELISA in (B). (**C**) Competing-risk survival analysis comparing γδ CAR-T treated C4-2B PSCA+ tumor-bearing mice to untreated and γδ GFP controls. (**D**) Table of statistical comparisons from competing risk survival analysis. n=7 mice/group. Statistical analyses performed by linear regression comparison of slopes or competing risk analysis.

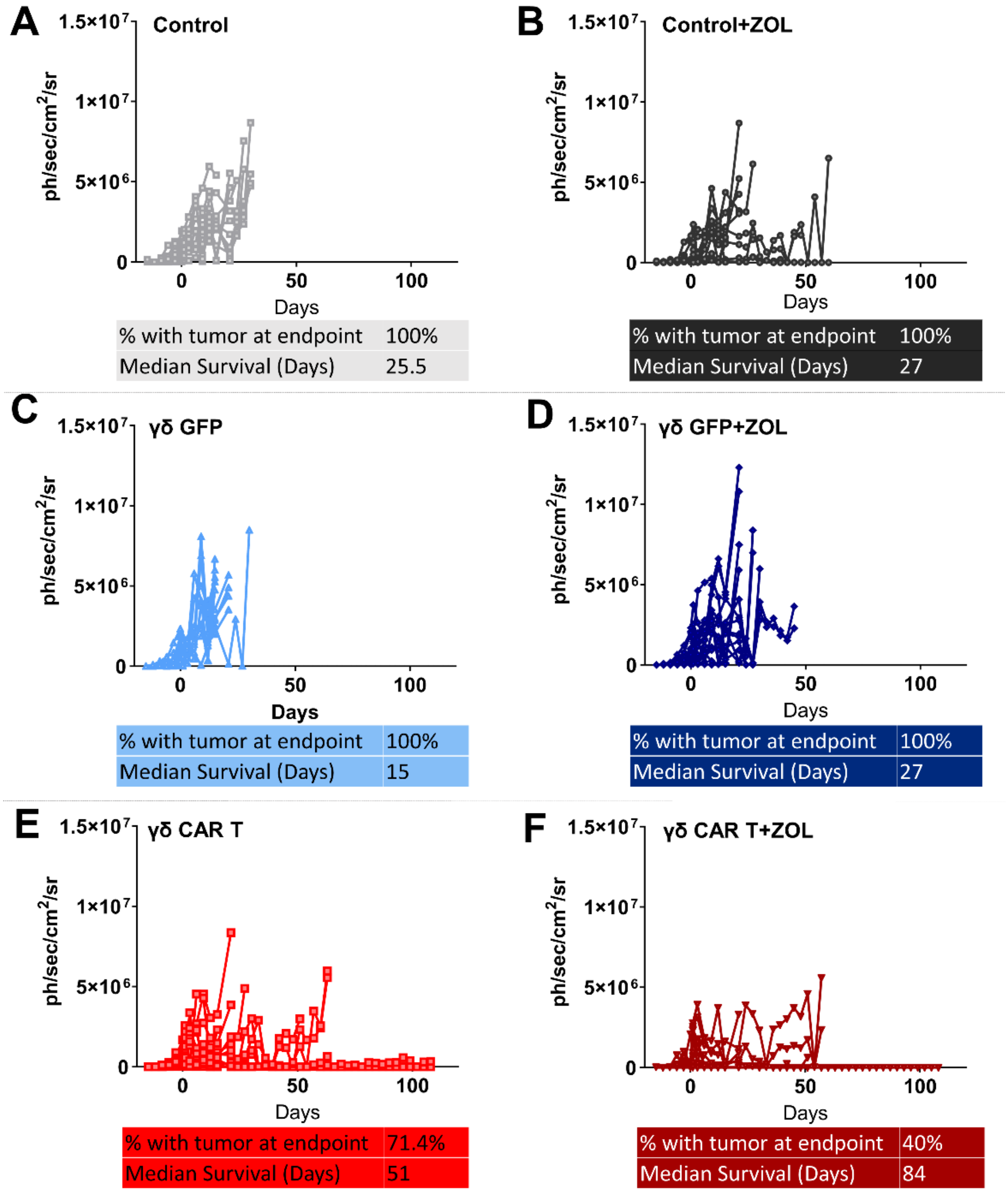

Fig. S10. Individual bioluminescence plots and median survival of intratibial prostate tumors. (**A** to **F**) Temporal luminescence imaging (ph/sec/cm2/sr) for each intratibial C4-2B PSCA+ prostate tumor in indicated treatment groups on equivalent y-axes scale. γδ CAR-T cells were delivered on Day 0. Percentage of mice with tumor at endpoint confirmed by comparison of luminescence and histology and the median survival (days) are indicated within inset tables. n=7 mice/group.

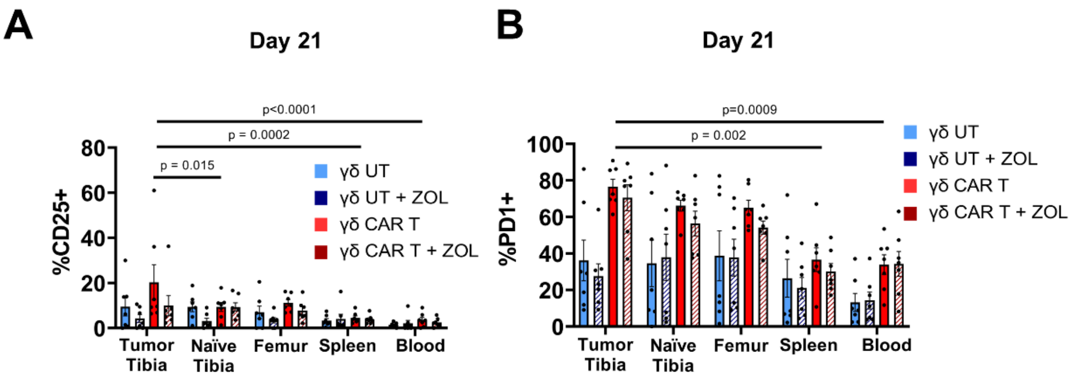

C

Comparison (CD25)	*Day 5 p-value*	*Day 21 p-value*
UT Tumor-bearing tibia vs. UT Tumor-naïve tibia	0.9858	>0.9999
UT Tumor-bearing tibia vs. UT Femur	0.9730	0.9096
UT Tumor-bearing tibia vs. UT Spleen	0.7644	0.2850
UT Tumor-bearing tibia vs. UT Blood	0.8377	0.1037
UT + ZOL Tumor-bearing tibia vs. UT + ZOL Tumor-naïve tibia	0.9998	0.9944
UT + ZOL Tumor-bearing tibia vs. UT + ZOL Femur	0.9942	0.9980
UT + ZOL Tumor-bearing tibia vs. UT + ZOL Spleen	0.8325	0.9999
UT + ZOL Tumor-bearing tibia vs. UT + ZOL Blood	0.8016	0.9369
CAR Tumor-bearing tibia vs. CAR Tumor-naïve tibia	<0.0001	0.0149
CAR Tumor-bearing tibia vs. CAR Femur	<0.0001	0.0557
CAR Tumor-bearing tibia vs. CAR Spleen	<0.0001	0.0002
CAR Tumor-bearing tibia vs. CAR Blood	<0.0001	<0.0001
CAR +ZOL Tumor-bearing tibia vs. CAR + ZOL Tumor-naïve tibia	<0.0001	0.9997
CAR +ZOL Tumor-bearing tibia vs. CAR + ZOL Femur	0.0001	0.9383
CAR +ZOL Tumor-bearing tibia vs. CAR + ZOL Spleen	<0.0001	0.2964
CAR +ZOL Tumor-bearing tibia vs. CAR + ZOL Blood	<0.0001	0.1494

Comparison (PD1)	*Day 5 p-value*	*Day 21 p-value*
UT Tumor-bearing tibia vs. UT Tumor-naïve tibia	0.3168	0.9998
UT Tumor-bearing tibia vs. UT Femur	0.4097	0.9980
UT Tumor-bearing tibia vs. UT Spleen	0.8863	0.8034
UT Tumor-bearing tibia vs. UT Blood	0.8495	0.1358
UT + ZOL Tumor-bearing tibia vs. UT + ZOL Tumor-naïve tibia	0.6019	0.7623
UT + ZOL Tumor-bearing tibia vs. UT + ZOL Femur	0.3484	0.7708
UT + ZOL Tumor-bearing tibia vs. UT + ZOL Spleen	0.9870	0.9428
UT + ZOL Tumor-bearing tibia vs. UT + ZOL Blood	0.9124	0.5807
CAR Tumor-bearing tibia vs. CAR Tumor-naïve tibia	0.9684	0.7670
CAR Tumor-bearing tibia vs. CAR Femur	0.7601	0.7035
CAR Tumor-bearing tibia vs. CAR Spleen	0.0006	0.0021
CAR Tumor-bearing tibia vs. CAR Blood	<0.0001	0.0009
CAR +ZOL Tumor-bearing tibia vs. CAR + ZOL Tumor-naïve tibia	0.8638	0.5323
CAR +ZOL Tumor-bearing tibia vs. CAR + ZOL Femur	0.9731	0.3989
CAR +ZOL Tumor-bearing tibia vs. CAR + ZOL Spleen	0.0100	0.0018
CAR +ZOL Tumor-bearing tibia vs. CAR + ZOL Blood	<0.0001	0.0059

Fig. S11. Activation phenotype of γδ T cells cells *in vivo* at day 21 after ACT. (**A**) Expression (%) of CD25 in γδ T cells at day 21 after adoptive cell therapy (ACT) in the different locations. (**B**) Expression (%) of PD-1 in γδ T cells at day 21 after adoptive cell therapy (ACT) in the different locations. Results depicted as mean ± SEM of n=6 mice/time point in two different experiments. Statistical analyses performed by 2-way ANOVA. Significance with respect to the CAR group in the different locations is shown. (**C**) p-values of the comparisons among the different groups in experiments shown in Figure 7 and Figure S11.

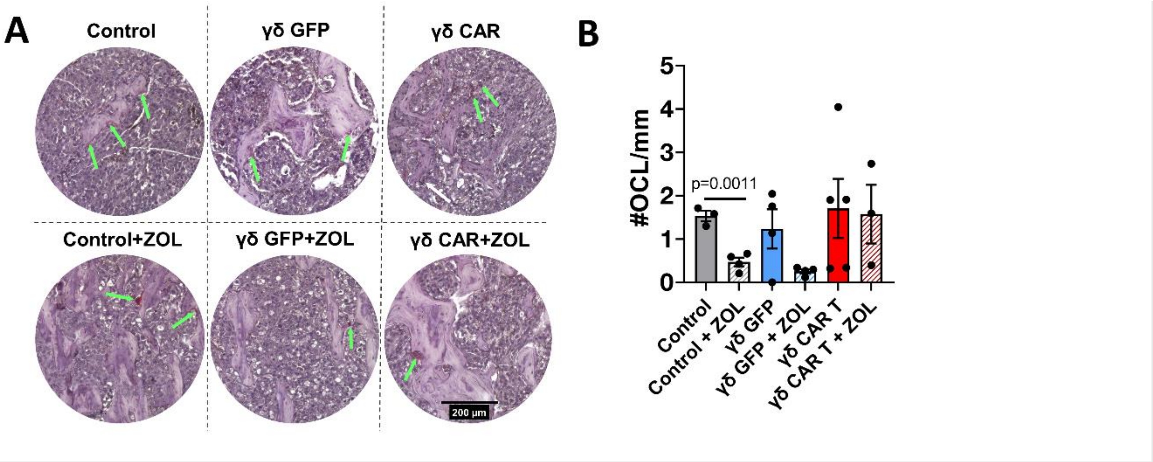

Fig. S12. Quantification of TRAcP+ cells in intratibial prostate tumors. (**A**) Representative images of tartrate resistant acid phosphatase (TRAcP) stained paraffin embedded tibia sections to visualize osteoclasts. Light pink represents bone, dark pink tumor, and dark red staining osteoclasts (indicated with green arrows). (**B**) Number of osteoclasts per mm of tumor-lining bone were quantified from micrographs using ImageJ. n=3-5. Each dot represents a sample. Statistical analyses performed by unpaired t-test between independent groups.

Table S1. Number of samples and tissue type in the tumor microarrays.

Tissue type	Bisphosphonate treated			Bisphosphonate naive			Total samples		
	Number of patients	Number total of samples	Number of samples used for analysis	Number of patients	Number total of samples	Number of samples used for analysis	Number of patients	Number total of samples	Number of samples used for analysis
Bone	18	67	33	19	78	31	37	145	64
Lung	4	11	11	6	18	16	10	29	27
Lymph Node	8	27	24	9	30	28	17	57	52
Liver	10	30	28	17	51	42	27	81	70
